# Multicriteria models provide enhanced insight for siting US offshore wind

**DOI:** 10.1093/pnasnexus/pgaf051

**Published:** 2025-03-04

**Authors:** Rudolph Santarromana, Ahmed Abdulla, M Granger Morgan, Joana Mendonça

**Affiliations:** Department of Engineering and Public Policy, Carnegie Mellon University, Pittsburgh, PA 15213, USA; Center for Innovation, Technology, and Policy Research (IN+), Department of Engineering and Management, Instituto Superior Técnico—Universidade de Lisboa, Lisbon 1049-001, Portugal; Department of Mechanical and Aerospace Engineering, Carleton University, Ottawa, ON, Canada K1S 5B6; Department of Engineering and Public Policy, Carnegie Mellon University, Pittsburgh, PA 15213, USA; Center for Innovation, Technology, and Policy Research (IN+), Department of Engineering and Management, Instituto Superior Técnico—Universidade de Lisboa, Lisbon 1049-001, Portugal

**Keywords:** offshore wind, sustainability, siting, renewable energy, competing interests, Physical Sciences > Sustainability Science

## Abstract

Offshore wind can be a key contributor to energy system decarbonization, but its deployment in certain regions has been slow, partly due to opposition from disparate interests. Failure to sufficiently address the concerns of external stakeholders could continue to hamper deployment. Here, we use a multi criteria model to assess all possible sites in a 2 km × 2 km grid of all potential locations in continental US federal waters, contrasting the perspectives of developers and other stakeholders. Our model elucidates how developers and policymakers could better approach future deployment. First, while developers prefer larger plants, we find that these facilities are more fragile—they are sensitive to location, and their impacts are more uncertain than smaller plants. Second, there is 600 GW of capacity where both developer and stakeholder interests align—developing these locations should be prioritized. Third, there are few areas on the US West Coast where developer and stakeholder preferences align, suggesting a need to reduce stakeholder–plant interactions or locate facilities in deeper waters than current technology allows.

**Significance Statement**

Project developers and other stakeholders often have divergent preferences with respect to offshore wind (OSW) farms. This paper suggests how to find sites and develop projects that reconcile the two perspectives and identify strategies to make project design and siting decisions more successful. The results contribute to the theoretical and policy issues involved in OSW siting using a spatial multi criteria analysis. They also enrich our understanding of how sociotechnical modeling approaches can improve the prospects of low-carbon energy project deployment. These could help inform siting and design decisions that accommodate community perspectives.

## Introduction

Large economies like China (1), the European Union (2), and the United States (3) view offshore wind (OSW) energy as a key contributor to deep decarbonization. However, implementation in the United States has been slow. While the United States set a target of 30 GW of OSW by 2030, as of the end of 2023, not a single commercial OSW project had been fully commissioned in federal US waters (4). The United States has >52 GW in the development pipeline (4), but past US experience raises doubts about how much of this will be deployed.

Stakeholder and financial interests have played a strong role in early US project development. The Cape Wind project was to be the first commercial OSW project in US federal waters, securing a lease in 2010 (5). The leaseholder relinquished the rights to build the plant in 2017 after dozens of legal cases were brought, reflecting concerns from coastal residents (1, 5, 6). The Morro Bay wind energy area in California faced scrutiny during its early planning stages from the US Navy, which claimed that part of the site would interfere with their activities (7). A legal case against Vineyard Wind argued that building the plant should not take precedence over other marine interests and activities like fishing, navigation, and the ecological environment (8). More recently, even projects with power purchase agreements in place elected to pay penalties and withdraw from their contracts, citing financial reasons (9, 10). As this brief history makes clear, project developers must balance many concerns in their decision-making. Figure S1 summarizes this record of unsuccessful US deployment.

Project design choices impact techno-economic, environmental, ecological, and spatial concerns. The OSW market has moved toward ever-larger plants and turbine sizes (4) because studies of project economics show that larger plants and turbines tend to reduce the cost of energy (11). However, experience also suggests that larger projects can result in cost overruns and schedule slippage (12), and that the adoption of smaller scale and modular deployments may accelerate adoption (13, 14). Planning and balancing the needs and uses of marine areas is important for sustainable energy development, but today the United States has no formal process for marine spatial planning (15). Better, systematic involvement and consideration of stakeholder concerns, as well as better communication of stakeholder benefits could improve the management of marine resources, areas, and water resource management policies (16).

Prior efforts to quantify the trade-offs involved in wind energy installations have considered visual, tourism/recreation, environmental/ecological, and industry impacts. Public concerns and perceptions have been studied as a trade-off for OSW siting (17, 18). Studies in Scandinavia (19, 20) and the United States (21) find that households are willing to pay to reduce visual disamenity from wind plants. Other assessments have considered plant impacts on tourism (22–24) and coastal recreation (25, 26). The value of reducing ecological and visual impacts of wind farms was assessed by Börger et al. (27). With respect to the impacts on shipping and fishing industries, Samoteskul et al. (28) find that the cost savings to OSW installations by siting closer to shore are greater than the increased costs due to vessel re-routing; and Hoagland et al. (29) quantify economic and welfare losses to the fishing industry realized by an OSW site. Another major theme in the literature is how social acceptance impacts the technology diffusion of renewable energy (30). Experience with social resistance to wind energy in North America (31, 32) and a review of social impacts from OSW in the United States and Europe suggests that a clear, equitable, and transparent decision process is a key to successful project implementation (33).

These multiple factors suggest that a spatial multi criteria analysis should be used in selecting OSW sites (34). Here, we build such a model that makes three novel contributions. First, we explore the impacts of combinations of sites, technologies, and plant sizes on certain criteria, developing the largest alternative space for an energy siting study of this kind. Second, we contrast alternatives that have attractive techno-economics with those that address a broad range of concerns that may be raised by environmentalists, coastal residents, fishing interests, and others who, in contrast to developers, we collectively term “external stakeholders,” or for simplicity, “stakeholders.” We employ this simplified distinction while acknowledging that developers are also stakeholders of a project and may not only be techno-economic optimizers. Similarly, stakeholders may not only wish to minimize ecological, industry, aesthetic, and environmental impacts—which includes the desire to maximize clean energy generation, classified as a benefit of building plant alternatives in this paradigm. We compare the best alternatives in each paradigm (developer vs. stakeholder) to identify where the two paradigms align and deem these to be “consensus” areas. Third, we point investors and policymakers to alternatives that score highly under both perspectives—therefore, more likely to succeed—and suggest how government incentives could encourage successful implementation. Our model uses publicly available datasets and representative weight profiles as used in prior work (35).

Prior multi criteria models for OSW siting have assessed a limited and discrete set of alternative plants, ignored the impact of plant size, and failed to compare alternative technological paradigms to not building a plant (36–38). Only two studies have used multi criteria methods to assess OSW site alternatives in a US domain (37, 39). Technical criteria (wind speed, proximity to shore, and water depth) are sometimes the only criteria considered to identify the suitability of proposed alternatives (40). Economic and social criteria (capital costs, revenues, proximity to shipping lanes, employment, and marine life impacts) are also accounted for in other studies on a limited set of alternatives (36–38). When studies have used experts to identify weights on the metrics, they are either limited in number or comprise a set of experts from the OSW industry (34, 36), even though stakeholders, including local communities, have considerable agency over proposed projects. A few studies develop a fuller perspective over an entire jurisdiction's territorial waters; however, the resulting suitability maps are binary (41), or only consider energy performance metrics (40), and no such suitability maps have been produced for the United States.

Here, we assess all possible sites in a 2 km × 2 km grid of continental US federal waters between 3 nautical miles (NM) and the 200 NM Exclusive Economic Zone (EEZ). We consider a range of technological value chains that include different plant and turbine sizes to demonstrate the potential importance of scale in assessing suitability, which is a topic of growing interest in the literature. The resulting suitability scores are compared with the status quo—a “No-Action” suitability score. Our method of aggregating the impacts of neighboring grid cells allows us to compare plants of varying capacity, component size, and footprint. The plant footprint is calculated considering the plant capacity and turbine size of each alternative (see Table S2 for the combinations and resulting size of the plants in terms of grid squares). Given the importance of disamenties caused by installations elucidated in prior work with choice experiments (27), we also quantify an OSW farm's visual impacts from shore, considering different turbine and plant sizes; evaluate the extent of disrupted seabed that OSW deployment might cause; identify and integrate submerged obstructions, including cables; and integrate vessel traffic density, fishing effort, and the estimated biodiversity in each cell. Although the literature most commonly focuses on the economies of scale associated with larger plants, these other impacts also tend to increase with plant size. To reflect this, there is a growing literature indicating that there may be benefits of smaller installations to achieve quicker decarbonization (13), and experience demonstrates that public preferences may sometimes be for smaller plants (42, 43). This work looks to quantify the trade-offs of different plant scales and technologies. It also provides a quantitative framework for monetary and impact assessments. Details of the technology value chains are in SI Note 2.

Building on recent OSW market analysis (44), our “baseline” facility is a 1,000 MW plant comprising 10 MW turbines, with fixed foundations at depths up to 60 m and moored floating foundations for depths between 60 and 1,300 m. Plants transmit power to shore via high-voltage cables. Results highlight when changes to this baseline case might lead to enhanced deployment prospects. Attributes considered (see Table S18) are: (i) total overnight capital cost; (ii) unit overnight capital cost; (iii) annual energy output; (iv) levelized cost of energy; (v) visual impact; (vi) impact on fishing; (vii) impact on marine life; (viii) impact on vessel traffic; (ix) disrupted seabed; and (x) obstructions. Annual energy output is the only attribute classified as a benefit, while the remaining attributes are classified as costs in the assessments that follow. The weight profiles have the same maximum score of 10.

These metrics represent a range of techno-economic outcomes and physical exposures between the plant and various social, conservation, environmental, and industry interests. However, the valuation of these impacts may go beyond the degree of exposure. For example, impacts on fishing activities and marine life can be classified based on the type of fish or species present in an area (45), and impacts on vessel traffic may depend on the type of vessel or shipping activity (28). Foundation type can also determine the extent of ecological impacts, as habitat loss can vary across fixed and floating types. However, other ecological effects are similar between monopile and floating types: please see (45, Table 9) for a detailed comparison of impacts with foundation type. We use the potential physical exposure here because the metrics can be computed given the plant assets and publicly available spatial datasets, and do not require value functions from each group of receptors. We apply “representative weighting profiles” constructed like those in previous work (35) (see Materials and methods). The simplicity of the method, therefore, allows analysis without surveying impacted stakeholders to elicit their values on the metrics and can be a tool used in early feasibility assessment stages of a project. We demonstrate the model's usefulness with observed data and provide insights on key decisions (size and sites) for project developers. In the Supplementary material, we consider several different representative weight profiles and conduct a sensitivity analysis.

## Results

### Integrating stakeholder considerations alters extent and location of OSW deployment

**Fig. 1.**
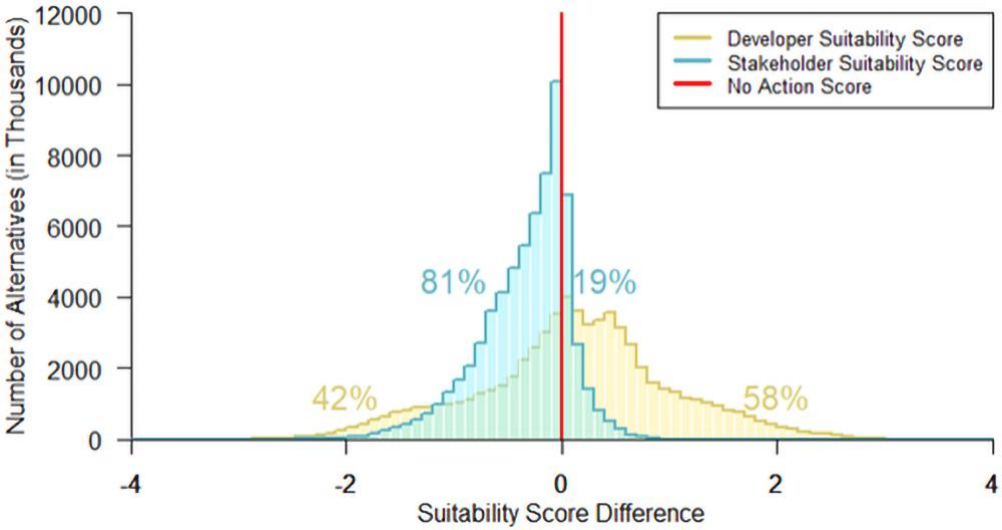
Histograms of the full alternatives space under both developer (yellow, flatter peak) and stakeholder (blue, sharper peak) paradigms illustrate how the attractiveness of OSW project alternatives changes from one to the other.

**Fig. 2.**
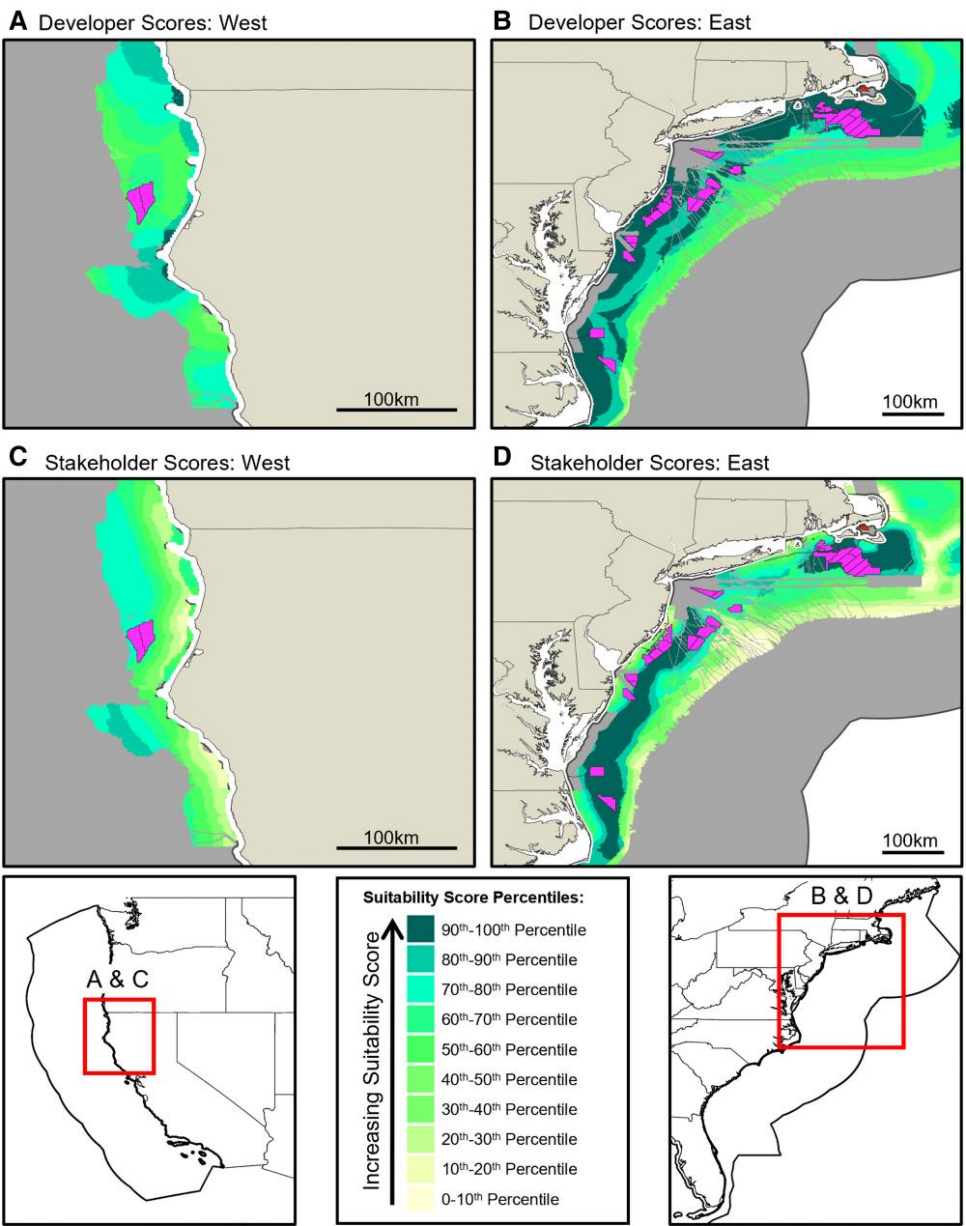
Suitability maps show the spatial distribution of site score quantiles for only the baseline facility (1,000 MW plant with 10 MW turbines and high-voltage direct current [HVDC] transmission). A and B) Developer suitability scores that tend to be higher nearshore; C and D) stakeholder suitability scores that tend to be higher away from shore—contrary to the developer scores. Uncolored areas are eliminated due to exclusion criteria; for example, the thin uncolored areas stretching from the coast are the locations of submarine cable exclusions; areas far from shore illustrate depth exclusions; and large areas nearshore show the locations of De Facto and other marine-protected areas (MPAs) (see Table 1). Existing wind development sites are also superimposed (irregular polygons).

The difference between an alternative's suitability score and the No-Action score is referred to as the “suitability score difference,” with a positive value defining “Build-Preferred” sites and a negative value defining “No-Build-Preferred.” The maximum theoretical score is 10, and therefore, a suitability score difference of ±1 represents ±10%, and an even greater proportion compared with the maximum alternative score. Most alternatives have scores within one point of the No-Action option (represented by a suitability score difference of zero). Therefore, while preferences are a continuum, we first characterize the results in a binary fashion, and assess the degree of difference in following results. From a developer perspective, 58% of the 66 million plant alternatives are Build-Preferred while 42% are No-Build-Preferred. In contrast, under the stakeholder paradigm, 19% of alternatives are build-preferred, whereas 81% are No-Build-Preferred. Figure 1 shows distributions of alternatives’ suitability score difference under each paradigm; Fig. 2 presents suitability maps of the East and West Coasts for the baseline facility under both paradigms. The distribution of the normalized scores for each metric across the entire alternatives space is given in Fig. S3.

These results (and others in the Supplementary material) suggest that developers would prefer to develop alternatives that will not be acceptable to a broad coalition of stakeholders, yielding opposition and potential delays or failure. This echoes findings from another study (46) and helps to explain why poorly chosen sites may fail in implementation, regardless of how promising they appear to developers. Suitability maps based on the developer paradigm (Fig. 2A and B) closely approximate maps of “economic potential” from a detailed assessment of US OSW sites (47). Sites closer to shore tend to score higher, with scores decreasing as one moves farther from shore. However, maps based on the stakeholder paradigm (Fig. 2C and D) tell a richer story in which attributes like visual impact, impact on fishing, impact on marine life, and impact on vessel traffic depress the suitability of nearshore locations, resulting in high-scoring stakeholder sites that do not abut the shore. While nearshore sites tend to score lower in the stakeholder paradigm, the results do not merely push sites as far offshore as possible. Some offshore locations are disfavored due to seabed disruptions, obstructions, or transmission losses that increase as plants move away from shore. The stakeholder paradigm comprises an analytical framework that enables these trade-offs to be expressed, discussed, and resolved by developers, analysts, and policymakers. The methods section explains the attribute weighting paradigms and presents other weighting paradigms considered in this analysis. For additional results, see Supplementary material.

We have considered existing and past OSW project proposals in the United States using the framework we develop here (see Table S13) and find that all project proposals that have submitted a construction and operations plan to the US Department of Interior as of March 2024 score highly in the developer paradigm. However, project suitability scores are depressed in the stakeholder paradigm. The results similar to those in Fig. 2 are found with several other plant value chains, such as those considering different turbine and plant sizes. Stakeholder suitability scores tend to be lower close to shore. Finally, it is notable that the canceled Cape Wind project (5) scores negatively in the stakeholder paradigm, meaning that a No-Build decision is preferred on that site. Hence, while our model is not intended to be predictive and additional site-specific assessments will be needed beyond the suitability scores we provide here, our model gives insight into the potential suitability of OSW plant proposals.

### Larger plants are more fragile from a stakeholder perspective

**Fig. 3.**
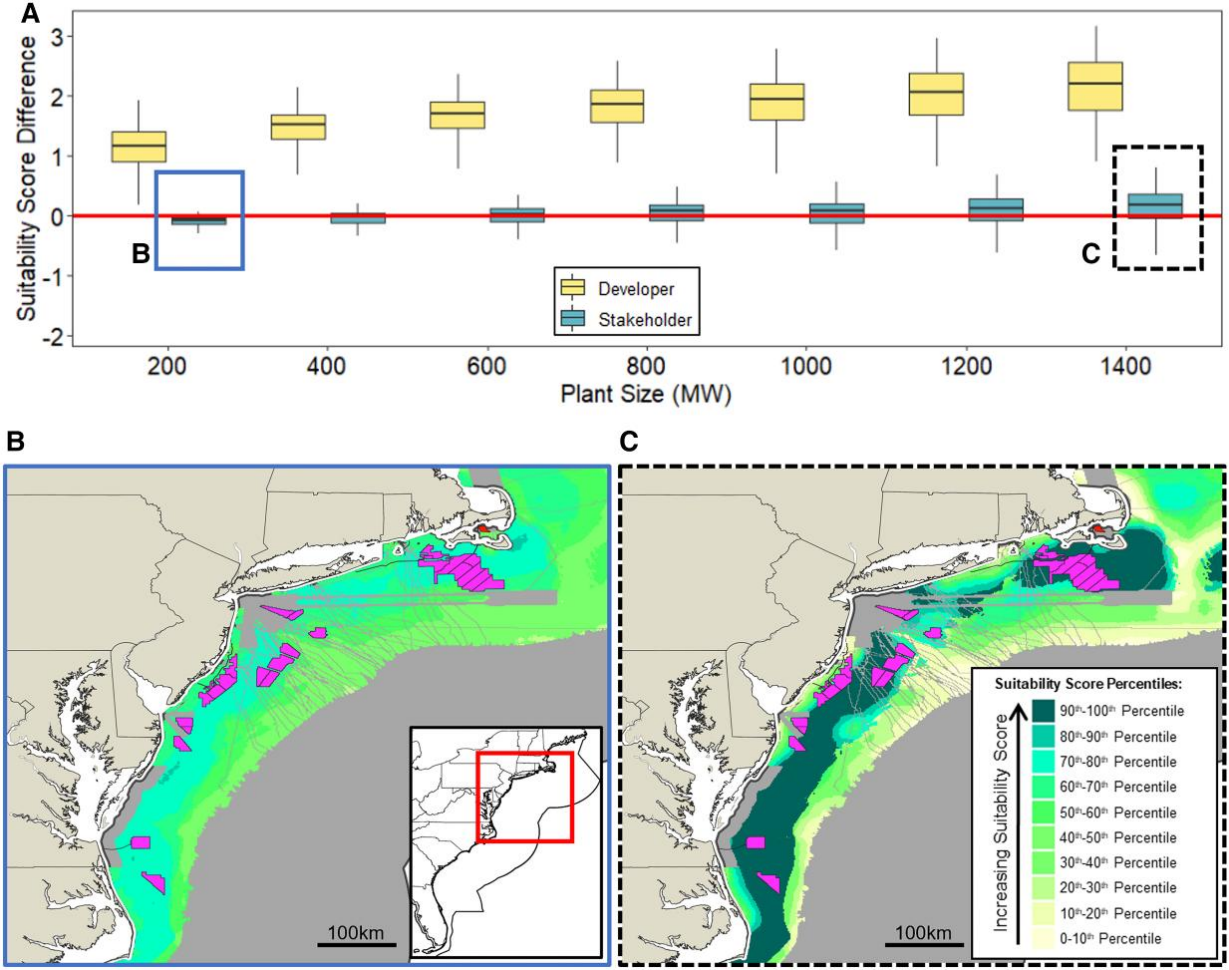
Plant size affects the distribution and variance of scores under both developer and stakeholder paradigms. A) Suitability score differences for various plant sizes under both paradigms shows variance increases with plant size. All plant sizes assume 10 MW turbine with fixed foundations and HVDC transmission. The maps illustrate the spatial distribution of quantiles across all plant scales for a B) 200 MW plant, showing that scores tend to be similar over a large area, and a C) 1,400 MW plant showing that drastic changes in scores are realized over small areas, indicating high stakeholder scores are largely determined by location. Existing wind development sites are also superimposed (irregular polygons).

Smaller plants typically impinge on fewer stakeholder interests than larger plants due to their smaller footprint. Since the largest plants accrue the largest impacts due to their environmental footprint and size, one would expect that they realize the lowest stakeholder suitability scores and are never favored. While the former is true, the latter is not: large plants also realize the best stakeholder suitability scores as illustrated in the boxplots in Fig. 3A—indicating that at some locations, plant impacts do not outweigh the benefits of lower unit cost and greater energy output. The suitability of large plants varies widely depending on location, and thus extreme care must be taken when siting large plants. Comparing the distribution of scores among the plant alternatives for the largest and smallest plant (see Fig. S5), one can see that the variance of nearly all criteria is wider for large plants. This phenomenon of high variance of impacts and energy output was also found with utility-scale solar in the United States (48). The accumulation of more assets in a larger plant and its interaction with a greater area leads to increased variance across nearly all criteria compared with smaller facilities. A similar effect of small variance for small plants is also found for other weight profiles assessed (Fig. S7).

The tighter distribution of scores for smaller plants under the stakeholder paradigm carries two implications. First, while it may be more difficult to identify sites that are unambiguously better than others, suitability scores for smaller plants are also more robust regardless of location, giving developers a certain amount of flexibility in locating their project—depicted graphically by the monotone distribution of colors in Fig. 3B, indicating similar scores are realized across sites. Small plant sizes could therefore help developers reduce the uncertainty arising from various types of stakeholder opposition. Second, larger plants are more fragile. These plants exhibit greater variance in scores, meaning that location matters greatly. Because only certain locations are suitable for deploying large plants (and since scores can fall sharply adjacent to very suitable areas demonstrated in Fig. 3C), developers who wish to build larger plants should pursue development in those locations, ceding some of the flexibility that smaller plants afford.

### There are areas where developer and stakeholder paradigms align

**Fig. 4.**
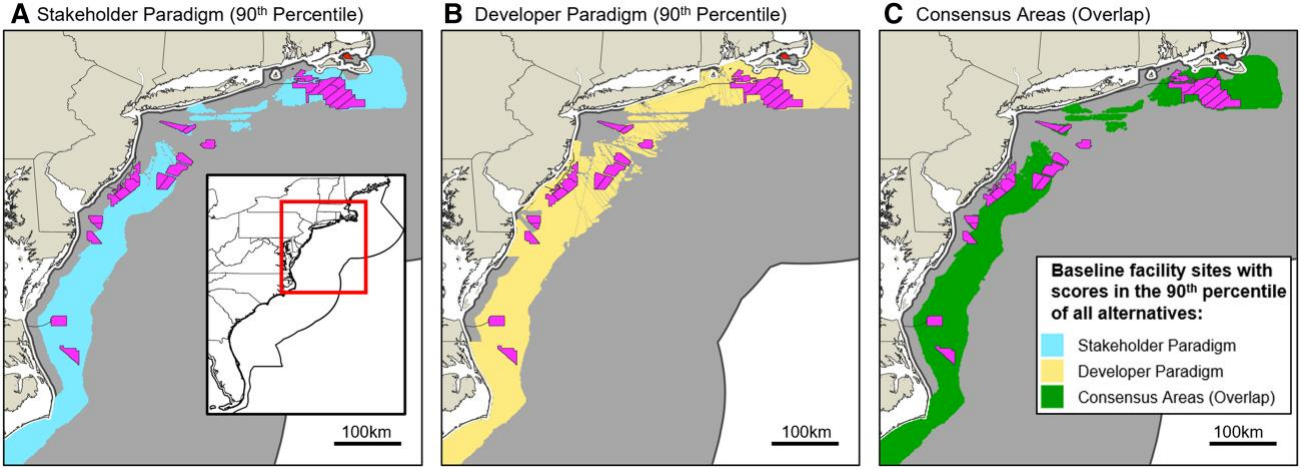
Baseline facility suitability scores in 90th percentile of all alternatives under the A) stakeholder and B) developer paradigms. C) Consensus areas where stakeholder and developer paradigms align show there is considerable overlap on the East Coast showcasing that consensus areas are vast, but some existing wind development areas are not within them. Existing wind development sites are also superimposed (irregular polygons).

Finding locations where both developer and stakeholder paradigms yield the highest suitability scores could be keys for more rapid and successful implementation of OSW projects. Given that many of the alternatives score close to the No-Action score (Fig. 1), we consider those that score in the top 10 percentile in each paradigm to be the least ambiguous in their preferability compared with the No-Action alternative. We then term alternatives that score at this level under both developer and stakeholder paradigms as “consensus” locations. Figure 4 presents these consensus locations on the East Coast.

Considering deployment only within the depth limits of fixed and moored technology (44, 49), this analysis finds enough consensus locations on the East Coast to deploy 600 GW of OSW (Fig. 4C). Many existing development areas on the East Coast fall within these consensus locations, though notably, some are not, and some are slightly closer to shore than the boundaries of consensus areas. On the West Coast, there are only enough consensus locations for 5 GW of OSW within existing depth constraints.

### Novel technologies are more expensive but could yield attractive West Coast sites

**Fig. 5.**
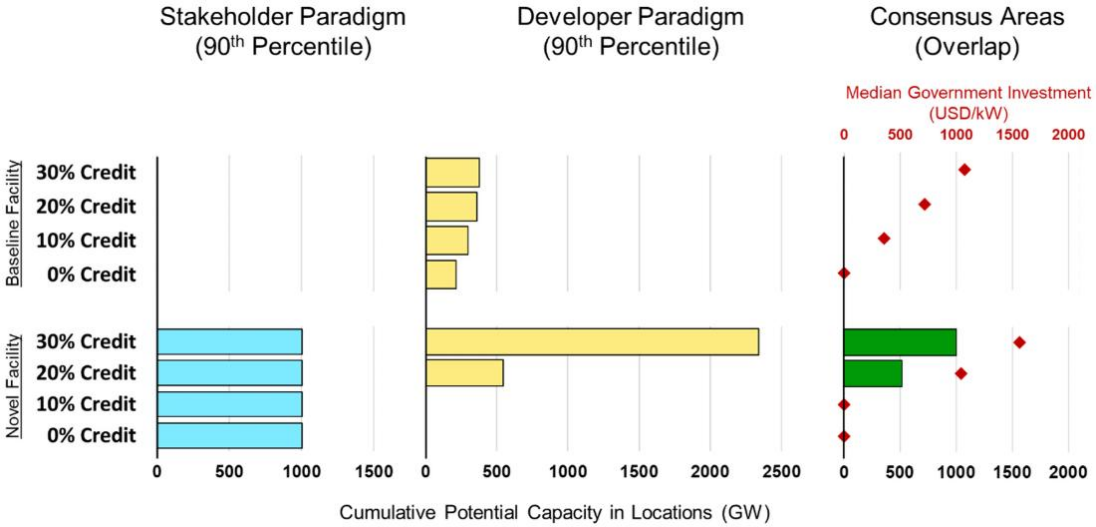
A comparison of the potential deployment capacity on the US West Coast of high-scoring (90th percentile of all alternatives) areas under the stakeholder paradigm (left), developer paradigm (middle), and consensus areas (right). Both the baseline facility (top half) and the novel DP + PtH facility (bottom half) are considered at different ITC amounts. The amount of government investment needed per kW is reported as points for the resulting consensus areas.

We find few consensus areas on the West Coast because of a lack of high-scoring sites under the stakeholder paradigm. This suggests that increasing a site's attractiveness under the stakeholder paradigm is necessary to unlock consensus areas along that coast. We investigate dynamically positioned (DP) floating OSW turbines (50–52) (see SI Note 4) and a power-to-hydrogen (PtH) transmission pathway—in which the OSW plant produces hydrogen through water electrolysis (53) that is delivered to shore via hydrogen ships instead of cables—as possible value chain alternatives. We refer to these technologies as “novel” to differentiate them from the baseline facility and denote that this combination of technologies has not yet been deployed. In addition to the advanced flexibility and economic benefits (54) that storage options may provide to OSW, these technologies eliminate seabed disruption from mooring chains, anchors, and cables, and largely remove plant depth limits—making deployment farther offshore possible and resulting in more high-scoring sites under the stakeholder paradigm on the West Coast. However, these technologies incur greater cost than traditional moored systems and involve lower end-to-end efficiency due to energy consumption by the DP thrusters and low electrolyzer efficiency (see Table S5), which depresses suitability scores under the developer paradigm. We find that this novel facility, combined with existing fiscal incentives, could help stimulate OSW deployment on the West Coast. Figure 5 shows the impact on the deployment of investment tax credits (ITCs) of varying amounts—up to 30%, which is in place thanks to the 2022 Inflation Reduction Act (55). This ITC would improve the cost-effectiveness of novel technologies, increasing their suitability scores under a developer paradigm and stimulating increased deployment in consensus areas.

Other forms of incentive may accomplish the same result, including a production tax credit or other subsidies that reduce the cost burden on developers, implying that a fiscal incentive could increase consensus areas for development by compensating for the reduced economic potential of stakeholder-preferred sites, or by enhancing the economic prospects of deploying novel technologies. On the West Coast, developing the novel DP + PtH systems in the resulting consensus areas would require a government investment of up to 1,600 USD/kW as seen in Fig. 5.

## Discussion

Here, we have presented results using a framework that compares representative developer and stakeholder paradigms. We have evaluated multiple weighting schemes beyond these paradigms, including the application of uniform weighting to all project attributes. Uniform weights generate an “improper linear model,” which not only requires fewer assumptions but has been shown to generate results that are as good or better than complex weighting schemes, including those derived through expert judgment (56, 57). Uniform weighting generates results that closely agree with the stakeholder paradigm presented here (see Fig. S6). We also find that increasing the weight placed on energy output increases the suitability scores of alternatives at all scales (Fig. S7C), implying that policies that increase the importance of deploying clean energy, such as those recommended by the European Commission (58), should be pursued.

As with all models, this work has some limitations. These include the possibility of including additional metrics such as job creation and pollution reduction as well as possible further disaggregation and weighting of sub-metrics (e.g. different vessel types, different fish stocks). Future efforts could also incorporate a temporal element into the study because costs will change over time, and thus, *when* a plant is built may be a key factor for future analysis. Here, we considered overnight costs, but financing plays a large role and could be included in future assessments. Financing costs can contribute half of an OSW project's levelized costs (49); smaller plants are less capital intense and would require less financing. Potential sources of error may come from cost estimates, physical estimates (volume and area), and energy output estimates, which may result in slight changes in the suitability scores. This is why we consider only the highest scores of alternatives when quantifying consensus areas. These and other matters, such as further exploration of alternative weighting strategies and the role of transparency in decision processes, are further discussed in the Supplementary material.

Our investigation of the effects of scale on suitability contributes to the conversation surrounding this topic. Recent work suggests that smaller, granular deployments and technologies are more conducive to growth and can thus be deployed more quickly in pursuit of urgent decarbonization (13, 14, 59). Preference has also been exhibited for smaller generation plants among local communities in southern Italy (42) and Germany (43) through choice experiments for terrestrial wind, mainly due to aesthetics. Our results show that smaller plants exhibit a tighter distribution of cumulative stakeholder impacts, demonstrating robustness to location. Smaller plant sizes also have better cost control as evidenced by a tighter distribution of capital costs (see Fig. S5H). The method applied here can be applied to different technologies and in different contexts to assess how the techno-economic suitability compares with a range of nonmonetary metrics that concern a broad coalition of stakeholders to find areas of overlap as done here.

Project development decisions regarding site, scale, and technology should not be made independently. As Fig. 3 illustrates, we find that areas that are suitable for some plant scales are not suitable for others. Our study is the first to incorporate the impact on the suitability of different OSW plant sizes. Furthermore, an analysis of a granular set of alternatives and sites as done here should be preferred to project-by-project stakeholder impact assessments. The former gives a fuller view of where cumulative impacts are the greatest, while the latter may result in developments being placed where the ability for stakeholders to petition decision-makers is weaker.

Our results suggest that the poor track record of deploying US OSW projects is unsurprising. Prior (and some current) site and design decisions align with the developer paradigm but not with the stakeholder one, offering an explanation for failed deployment. The implications for developers and policymakers are 3-fold. First, developers that have a strong preference for large projects should limit their attention to locations with higher scores under the stakeholder paradigm. To avoid failed deployment, policymakers should develop strategies that incentivize such choices. Second, while smaller plants yield higher unit and levelized costs, they might prove appropriate in more locations and institutional contexts than larger plants. Third, to facilitate development along the US West Coast and other coasts where depths increase rapidly, policymakers should provide research support and financial incentives to increase the viability of technologies that are deployed far offshore and score highly under the stakeholder paradigm. Our analysis shows that harnessing the large energy potential and decarbonization potential that OSW has to offer is possible, but developers should pursue projects that achieve both good techno-economic viability and lower stakeholder impacts—considering techno-economics alone in plant design is insufficient.

OSW holds the potential to increase energy independence and make a major contribution to decarbonizing the energy system. However, unless developers and policymakers become more cognizant of stakeholder concerns and adopt strategies that mitigate them, the potential for radically expanded deployment could be limited.

## Materials and methods

### Analysis domain setup

The geographic domain is the federal waters of the contiguous United States: all waters from 3 NM from the shoreline (9 NM from the shoreline for Texas and the Gulf Coast of Florida as per the Submerged Lands Act (60)) to 200 NM from the shoreline—the extent of the EEZ—of the continental US Alaska and Hawaii are not considered in this study because most of the US development activity has thus far been in the continental states, and some of the project sizes considered in this analysis may not be viable in these states. A 2 km × 2 km grid is established, and each grid square may contain one wind turbine, or one collector platform. QGIS is an open-source software used as the primary geographic software for this analysis. This grid spacing follows recommendations from a US Coast Guard study, which recommends 1 NM (1.85 km) spacing between turbines (61). There are 868,064 2 km × 2 km grid squares in the continental US federal waters.

The technological domain describes the value chain options of turbine size, station-keeping type, plant size, and transmission method (Table S14). A detailed description of each technology is provided in SI Note 2. These options were chosen based on data availability and to get a range of current and future technologies which may be used to deploy OSW. Novel technologies considered—DP station keeping and PtH transmission—offer a trade-off in increased costs, at a reduced physical presence (fewer obstructions and disrupted seabed).

There are 168 value chains possible given the combinations of options listed in Table S14. We further consider one No-Action option in which a plant is not developed. Considering any of these value chains centered in any of the grid squares, there are 146,702,816 plant alternatives (combinations of value chain options and locations) in this analysis.

### Grid square metric calculations

Grid square metrics consider the parameters of the value chain. The following metrics are computed for each value chain—some only pertain to a few options.

- Turbine visibility
- Volume of obstructions
- Disrupted seabed area
- Turbine annual energy
- Turbine asset cost
- Export system cost
- Export system efficiency

We compute the relevant metrics should either a turbine or collector platform be placed in the grid square. Upon calculating the plant metrics in the next step, only the metrics for the asset in the grid square are considered (see SI Note 11 for details).

### Plant-site metric calculation

A plant will require several turbines, occupy numerous grid squares, and may have different layouts. The final plant metrics should reflect the magnitude and footprint of the overall plant. As this is not a layout optimization study, we do not choose a single layout, but instead, combine the metrics of neighboring grid squares using the following process, carried out for the entire geographic domain (at each grid square).

1. The number of grid squares needed for the plant is determined as the discrete number of turbines needed given the plant and turbine size of the value chain with a ceiling function:
  $$n_{\mathrm{turbine}}=\frac{\mathrm{Plant}\;(\mathrm{MW})}{\mathrm{Turbine}\;(\mathrm{MW})}$$
2. An “influence area” (IA) is defined which considers the grid squares that may be part of the plant. This is done by taking all the grid squares within a maximum distance, *D*, from the collector grid square, *i*, computed as,
  $$D=\sqrt{n_{\mathrm{turbine}}}\;$$

this distance defines the side length (in number of turbines) of a square array given $n_{\mathrm{turbine}}$. A square array can be placed with the collector site at any corner and fit within the IA along with other plausible orientations of turbines (see example in Fig. S10). The IA therefore gives an approximation of which grid squares would likely be part of a plant with the collector at its centroid without requiring that a layout be chosen.

3. Within the IA, we consider five exclusion criteria where an asset may not be installed:
  (a) Existing cables: grid squares that have any submarine cables running through them are eliminated from the IA when the value chain has fixed foundations or moored turbines.
  (b) Depth limit: grid squares with depths >60 m for fixed foundation turbines (49), and depths >1,300 m for moored floating turbines (44) are eliminated from the IA.
  (c) MPA: grid squares that overlap with an established MPA are eliminated from the IA regardless of the technology used.
  (d) Shipping fairways: grid squares that overlap with an established shipping fairway are eliminated from the IA regardless of the technology used.
  (e) Cultural and heritage: grid squares that overlap a “De Facto” MPA (62) with a designated restriction on energy extraction are eliminated from the IA regardless of the technology used.

These exclusions are also applied to the collector grid square itself. If the collector grid square satisfies either of these criteria, the plant alternative is removed from the alternatives space. Some of these exclusions (like submarine cable presence) may instead require different design solutions or protections rather than being prohibited from building. Sixty-six million plant alternatives from the original 146 million remain after exclusions.

4. The remaining grid squares within the IA are used to compute the plant metrics, described in further detail below. Each metric is classified as either a benefit or a cost. If the number of remaining squares in the IA is less than the number of turbines needed for the plant, the plant alternative is removed from the alternatives space. If the number of remaining grid squares in the IA is greater than the number of turbines, some of the metrics are scaled by the number of “effective plants” remaining in the IA,
  (1) $$\;\mathrm{Effective}\;\mathrm{plants}=\;\frac{\mathrm{Number}\;\mathrm{remaining}\;\mathrm{grid}\;\mathrm{squares}\;\mathrm{in}\;\mathrm{IA}}{n_{\mathrm{turbine}}}$$

#### Overnight capital cost and unit overnight capital cost

The capital costs of the components are summed to get a plant overnight capital cost, reported as the overall cost and cost per installed MW of plant capacity. The details of cost assumptions and computations for the components of the plant (including the turbine, substructure, inter-array cables, onshore interconnection, export cable, and station keeping) are provided in SI Note 5.

#### Annual energy/H_2_ output

Estimated considering the losses in transmission to shore and conversion (in the case of hydrogen production).


$$\begin{array}{rl} E[\mathrm{Annual}\;\mathrm{energy}]\;= & \;\frac{\sum_{\mathrm{IA}}\;\mathrm{Annual}\;\mathrm{turbine}\;\mathrm{generation}}{\mathrm{Effective}\;\mathrm{plants}}\\ & \times \mathrm{Transmission}\;\mathrm{efficiency} \end{array}$$

$$\begin{array}{rl} E[\mathrm{Annual}\;H_{2}]\;= & \;\frac{\sum_{\mathrm{IA}}\;\mathrm{Annual}\;\mathrm{turbine}\;\mathrm{generation}}{\mathrm{Effective}\;\mathrm{plants}}\\ & \times \mathrm{Conversion}\;\mathrm{and}\;\mathrm{shipping}\;\mathrm{efficiency} \end{array}$$

#### Levelized Cost of Energy

The levelized cost of energy (LCOE) and levelized cost of hydrogen (LCOH) are computed as the net present value (NPV) of costs divided by the NPV of energy flows considering overnight capital expenditure (CAPEX) and annual operational expenditures (OPEX) which are estimated as a percentage of CAPEX incurred per year (see Table S7). A discount rate, *r*, equal to 5.2% is used (63).


$$\mathrm{LCOE}\left[\frac{\mathrm{USD}}{\mathrm{MWh}}\right]=\;\frac{\mathrm{NPV}\;\mathrm{of}\;\mathrm{costs}}{\mathrm{NPV}\;\mathrm{of}\;\mathrm{energy}\;\mathrm{flows}}=\frac{\sum_{t}\;\left(\frac{\mathrm{CAPE}X_{t}+\mathrm{OPE}X_{{}_{t}}}{{\left(1+r\right)}^{{}_{t}}}\right)}{\sum_{t}\;\left(\frac{E[\mathrm{Annual}\;\mathrm{energy}]}{{\left(1+r\right)}^{{}_{t}}}\right)}$$

$$\mathrm{LCOH}\left[\frac{\mathrm{USD}}{\mathrm{MWh}}\right]=\;\frac{\mathrm{NPV}\;\mathrm{of}\;\mathrm{costs}}{\mathrm{NPV}\;\mathrm{of}\;H_{2}\;\mathrm{flows}}=\frac{\sum_{t}\;\left(\frac{\mathrm{CAPE}X_{t}+\mathrm{OPE}X_{t}}{{\left(1+r\right)}^{t}}\right)}{\sum_{t}\;\left(\frac{E[\mathrm{Annual}\;H_{2}]}{{\left(1+r\right)}^{t}}\right)}$$

#### Visual impact

Expected number of visible turbines in the IA from shore is divided by the number of effective plants.


$$\mathrm{Visual}\;\mathrm{impact}=\;\frac{\mathrm{Visible}\;\mathrm{turbines}\;\mathrm{in}\;\mathrm{influence}\;\mathrm{area}}{\mathrm{Effective}\;\mathrm{plants}}$$

#### Impact on vessel traffic

Number of vessel transits in the IA before the plant is built divided by the number of effective plants. The measure used here is vessel transits in each grid square. Therefore, the same vessel transiting adjacent grid squares in the same IA is counted more than once. While this may seem like multiple counting of a single ship, in fact, it captures the intensity of the impact on traffic due to the development in the IA. For example, a vessel that transits only one grid square on the periphery of the IA would be less impacted than a ship that transits through the middle of the IA, crossing many squares.


$$\mathrm{Impact}\;\mathrm{on}\;\mathrm{vessel}\;\mathrm{traffic}=\;\frac{\sum_{\mathrm{IA}}\;\mathrm{Vessel}\;\mathrm{transits}}{\mathrm{Effective}\;\mathrm{plants}}$$

#### Impact on fishing

Hours spent fishing per year in the grid squares of the IA prior to building the plant, divided by the number of effective plants. The same rationale to address potential “multiple counting” for the impact on vessel traffic applies here.


$$\mathrm{Impact}\;\mathrm{on}\;\mathrm{fishing}=\;\frac{\sum_{\mathrm{IA}}\;\mathrm{Hours}\;\mathrm{of}\;\mathrm{fishing}}{\mathrm{Effective}\;\mathrm{plants}}$$

#### Impact on marine life

Number of species observed in the IA divided by the number of effective plants. The same rationale to address potential “multiple counting” for the impact on vessel traffic applies here.


$$\mathrm{Impact}\;\mathrm{on}\;\mathrm{marine}\;\mathrm{life}=\;\frac{\sum_{\mathrm{IA}}\;\mathrm{Marine}\;\mathrm{species}\;\mathrm{richness}}{\mathrm{Effective}\;\mathrm{plants}}$$

#### Disrupted seabed

Estimated as the disrupted seabed caused by the turbines and cabled transmission systems.


$$\begin{array}{rl} \mathrm{Disrupted}\;\mathrm{seabed}\;= & \;\frac{\sum_{\mathrm{IA}}\;\mathrm{Disrupted}\;\mathrm{seabed}\;\mathrm{from}\;\mathrm{turbines}}{\mathrm{Effective}\;\mathrm{plants}}\\ & +\mathrm{Disrupted}\;\mathrm{seabed}\;\mathrm{from}\;\mathrm{cables} \end{array}$$

#### Obstructions

Obstructions from turbines in the IA divided by the number of effective plants.


$$\mathrm{Obstructions}=\;\frac{\sum_{\mathrm{IA}}\;\mathrm{Obstructions}}{\mathrm{Effective}\;\mathrm{plants}}$$

#### Cost exclusions

There are limits to the costs that developers might pay to deploy a plant. Some of the alternatives may surpass observed energy deployment costs. The maximum unit capital cost to deploy a renewable energy plant observed in historical data is 11,000 USD/kW (64), and the maximum LCOE observed is 360 USD/MWh (65). Alternatives that realize values greater than either of these are removed from the alternative space. A summary of all the exclusions applied to the alternatives space is seen in Table 1.

**Table 1.** Summary of exclusion criteria and for which value chain the criterion applies.

Where the exclusion applies	Criterion	For which value chain the exclusion is applied
Eliminates grid squares from the IA	Submarine cable present = 1	Moored and fixed
	Mean depth > 1,300 m	Moored
	Mean depth > 60 m	Fixed
	Marine-protected areas = 1	All
	Shipping fairway present = 1	All
	De Facto marine-protected areas = 1	All
Eliminates plant alternatives from the alternatives space	LCOE > 360 USD/MWh	All
	Unit OCC > 11,000 USD/kW	All

### Suitability score calculation

#### Normalize metrics

The metrics are normalized using a linear normalization method:


(2) $$Z_{ij}=\{\begin{array}{c} \begin{array}{cc} \frac{x_{ij}}{\max_{i}(x_{j})}, & \mathrm{if}\;j\;\mathrm{is}\;a\;\mathrm{benefit}\\ 1-\frac{x_{ij}}{\max_{i}(x_{j})}, & \mathrm{if}\;j\;\mathrm{is}\;a\;\mathrm{cost} \end{array} \end{array}\;\;\;\;$$

The normalized score, *Z*, for alternative *i* in criterion *j* is given a value of 0 to 1, where 1 is the most desirable score for the criterion. $x_{ij}$ is the numeric value of alternative *i* in criterion *j*, and $\max_{i}(x_{j})$ is the maximum value across the alternative space.

#### Weight profiles

The weight profiles (Table 2) are developed considering different representative preference profiles—similar to Klein and Whalley (35)—that place uniform weight on the metrics that are of concern to developers and impacted stakeholders. One benefit of generating weight profiles in this way is that with a national scope, eliciting weights from decision-makers or communities begs the question of “which community or set of individuals should evaluate the weights on criteria”? Different individuals and communities weigh decision criteria differently as noted in a National Academies report (66).


(3) $$0\leq w_{k}<\mathrm{Maximum}\;\mathrm{weight}$$

(4) $$w_{p}=\frac{\mathrm{Maximum}\;\mathrm{weight}-w_{k}}{n_{p}}$$

(5) $$w_{o}=0$$

**Table 2.** Metrics, weights, and no-action decision suitability scores under different weighting paradigms.

Metric	Main interested party	Unit	Cost or benefit	No-action metric value	Normalized no-action metric^a^	Developer weight	Stakeholder weight
*j*				$x_{NA,j}$	$Z_{\mathrm{NA},j}$	$w_{j}$	
Overnight capital cost	Developer	MUSD	Cost	0	1	2.5	0
Unit overnight capital cost	Developer	USD/MW	Cost	0	1	2.5	0
Annual energy/H_2_ output	Developer, stakeholder	MWh/year or tH_2_/year	Benefit	0	0	2.5	10/7
LCOE/LCOH	Developer	USD/MWh or USD/kgH_2_	Cost	—^b^	0	2.5	0
Visual impact	Stakeholder	Turbines/plant	Cost	0	1	0	10/7
Impact on fishing	Stakeholder	Hours/year	Cost	0	1	0	10/7
Impact on marine life	Stakeholder	Species/plant	Cost	0	1	0	10/7
Impact on vessel traffic	Stakeholder	Vessel transits/plant	Cost	0	1	0	10/7
Disrupted seabed	Stakeholder	km^2^ of seabed/plant	Cost	0	1	0	10/7
Obstructions	Stakeholder	m^3^ of obstructions/plant	Cost	0	1	0	10/7
No-action suitability score (out of 10 possible): $\sum w_{j}Z_{\mathrm{NA},j}$						5.0	8.6

The maximum possible score under each set of weights is equal to 10.

^a^The normalized metrics are computed as defined in Eq. 2, considering whether the metric is a cost or benefit.

^b^Undefined.

A key metric, k, may be identified and assigned to a single key metric of concern. The preferred metrics, p, each have an equivalent portion of the maximum weight (Eq. 4), and the remaining metrics, o, are given a value of zero.

A uniform weight profile assigns an equal value of one to each of the criteria; it was first proposed by Dawes and Corrigan (56) and has since been used in renewable energy site selection models (67). Our developer weight profile assigns equal weights to costs and output. Our stakeholder weight profile assigns equal weights to impacts and disamenities. All the metrics are classified as “costs” except annual energy generation, which is classified as a “benefit.” The weight profiles have the same maximum score of 10.

#### Suitability scores using simple additive weighting

A suitability score is computed using a simple additive weighting method:


(6) $$\mathrm{Scor}e_{i}=\sum w_{j}Z_{ij}$$

in which the normalized metrics are multiplied by their respective weights in the chosen weight profile and summed to give the alternative suitability score (vector dot product). The model is constructed such that a higher suitability score is the preferred alternative. A linear weighting model is applied here (as opposed to entropy weighting, for example) as it is a widely used method, results in easier interpretability, and does not require great computational demands for such a large dataset. Unlike data-driven weighting methods that derive weights from the underlying data's statistical properties, the weighting paradigms are constructed to establish distinct and divergent perspectives for comparison. In future work, other weighting methods, including elicited weights, can be integrated into this framework. To compare the plant alternatives to maintaining the status quo and not developing a plant, we quantify the score of a No-Action decision. The suitability scores of alternatives are compared with the No-Action score in that weight profile to identify whether the plant is Build-Preferred or No-Build-Preferred, in which the alternative suitability score is greater than or less than the No-Action score, respectively. Results of the additional weight profiles not in the main text are included as additional results the SI Note 8.

## Supplementary Material

pgaf051_Supplementary_Data

## Data Availability

The R code used in this analysis is provided in the following DOI: https://doi.org/10.5281/zenodo.11245334. The Materials and methods section describes each step, and further details of the method are provided in SI Note 11.
