# Supplementary material for: Multicriteria models provide enhanced insight for siting US offshore wind
Source: PNAS Nexus. 2025 Mar 4;4(3):pgaf051. doi: 10.1093/pnasnexus/pgaf051 (PMC11879181; doi:10.1093/pnasnexus/pgaf051)
Supplement: pgaf051_Supplementary_Data [file pgaf051_supplementary_data.pdf]

Supporting Information for

Multi-criteria models provide enhanced insight for siting U.S. offshore wind

Rudolph Santarromana[a,b,*], Ahmed Abdulla[c], M. Granger Morgan[a], Joana Mendonça[b]

a Department of Engineering and Public Policy, Carnegie Mellon University, Pittsburgh, PA 15213, USA

b Department of Engineering and Management, Instituto Superior Técnico - Universidade de Lisboa, Lisbon, 1049-001, Portugal

c Department of Mechanical and Aerospace Engineering, Carleton University, Ottawa, ON K1S 5B6, Canada

* Corresponding Author: rsantarr@andrew.cmu.edu

This PDF file includes:

Supporting text
Figures S1 to S10
Tables S1 to S19
SI References

Note 1. Summary of U.S. Offshore Wind Capacity Development

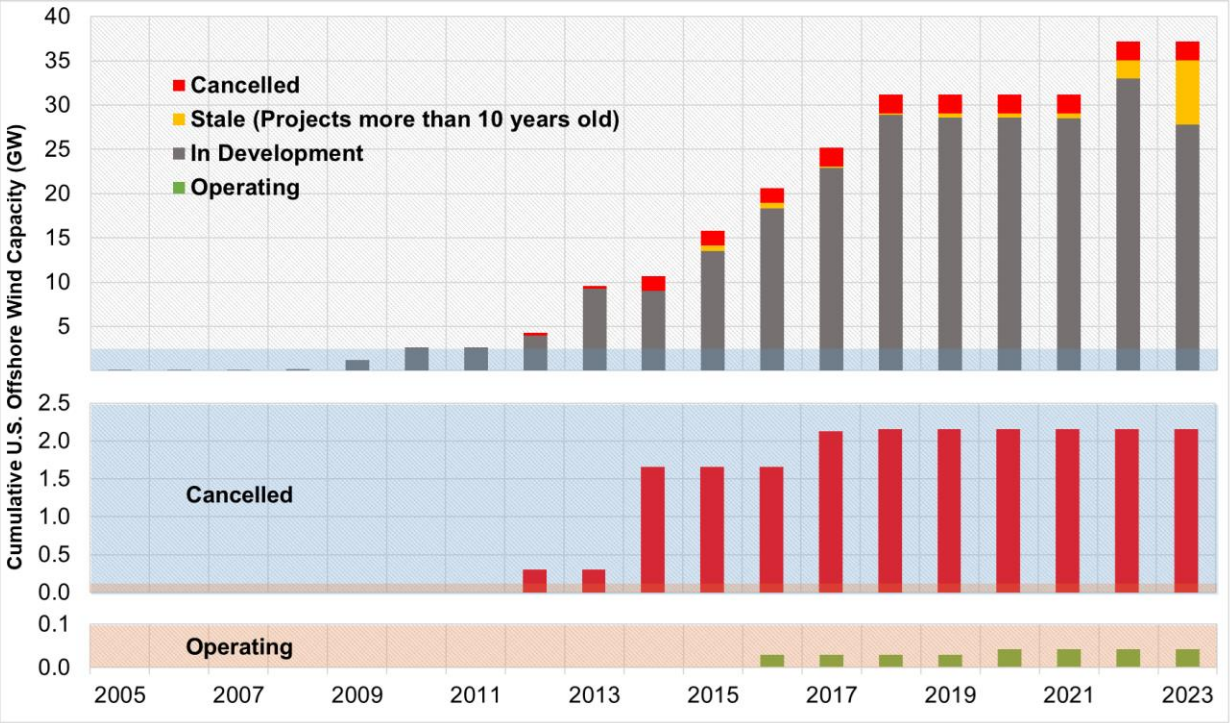

Figure S1. U.S. leased offshore wind capacity potentials based on status (1–9).

Note 2. Value Chain Technology Descriptions

Turbine and Plant Sizes. Four turbine types are considered as options in this analysis as described in Table S1. These are reference turbine specifications and represent a range of commercial or next-generation turbine capacities.

Table S1. Turbine specifications and dimensions

Turbine Rated Power [MW]	5	8	10	15
Hub Height [m]	90	110	119	150
Tower Base Diameter [m]	6	7.7	8.3	10
Blade Length [m]	61.5	82	96	117
2 km spacing in rotor diameters [-]	16.3D	12.2D	10.4D	8.5D
Cut-in Wind Speed [m/s]	3	4	4	3
Rated Wind Speed [m/s]	11.4	12.5	10.5	10.6
Cut-out Wind Speed [m/s]	25	25	25	25
Source	(10)	(11)	(12)	(13)

These turbine sizes are deployed in plants of various sizes ranging from 200 MW to 1,400 MW in this study. A discrete number of turbines is deployed for the plant, therefore, in certain instances, the plant nameplate capacity with some turbine sizes may be more than the nameplate capacity of others as shown in Table S2.

Table S2. Actual nameplate capacity for some turbine and plant size combinations in this analysis. Number of turbines required is given in parentheses.

		200	600	1000	1400
**Turbine Size (MW)**	**5**	200 (40)	600 (120)	1000 (200)	1400 (280)
	**8**	200 (25)	600 (75)	1000 (125)	1400 (175)
	**10**	200 (20)	600 (60)	1000 (100)	1400 (140)
	**15**	210 (14)	600 (40)	1005 (67)	1410 (94)

Station-keeping Types. Three station-keeping types are considered. A monopile foundation is the only fixed foundation type in this analysis in which a cylindrical substructure that is embedded in the seabed supports the wind turbine above the water surface. Monopiles are considered for depths up to 60 m (14). Two types of station-keeping are considered for floating foundations—moored and dynamic positioning (DP). A floating semisubmersible platform as described in Allen et al. (2020) is used for all floating turbines (15). A schematic and the dimensions of the floating semisubmersible are given in Figure S2.

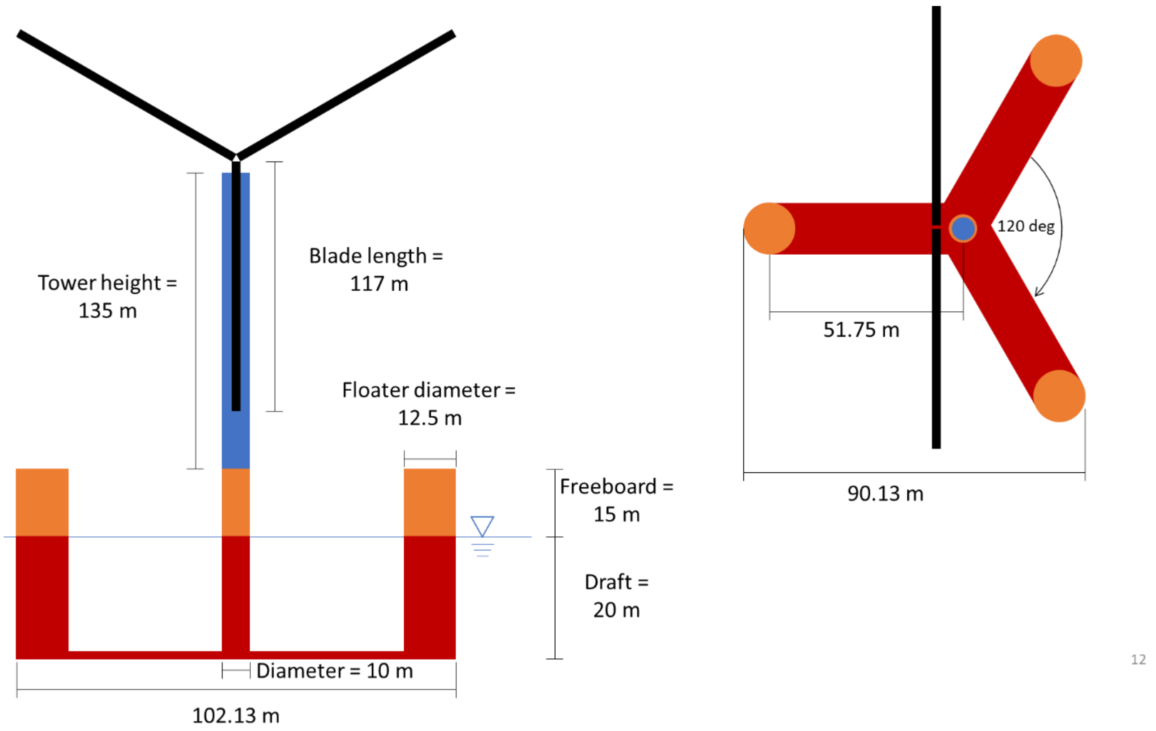

Figure S2. Dimensions of floating substructure used, adapted from (15).

A mooring system uses catenary mooring chains and drag embedment anchors to keep a floating turbine in position. A DP system uses thrusters mounted on the underside of the floating platform to counteract the environmental forces from wind, wave, and currents to keep the turbine in position(16, 17). The DP system consumes some of the energy output from the generating turbine, and therefore the net energy generation from the turbine is decreased when deployed with DP.

Transmission Types. Two transmission types are considered in this analysis. A high voltage direct current (HVDC) system is considered as the most likely candidate for high-capacity transmission for offshore wind (OSW) plants in the near term. In an HVDC transmission system, the energy generated at the turbines is collected at a single collector platform using inter-array cables between turbines and the platform, where an offshore substation is located. At the offshore substation (OfSS) a step-up transformer increases the voltage of the system. At the OfSS, the electricity is also converted from alternating current to direct current. Subsea high voltage transmission cables then transport the electricity from the OfSS to shore. The cables are typically buried in the seabed and require some area of buffer around the cable during service to prevent damage to cables and potential harm to flora and fauna in the vicinity of the cable. The cables then make landfall on the coast where they arrive at an onshore substation (OnSS). At the OnSS, the electricity is converted back to alternating current, and the voltage may be reduced. From the OnSS, the electricity is distributed through the existing transmission system. Specifics on the cables considered in the analysis to compute transmission losses for HVAC and HVDC systems are provided in the Table S3 and Table S4 below, taken from Xiang et al. (2021) (18):

Table S3. HVAC cable parameters considered in the analysis (18).

Voltage (kV)	Size (mm^2)	Resistance (Ω/km)	Nominal Current (A)	Capacitance (F/km)	Cost (GBP/km)
132	500	4.93E-02	739	1.92E-07	6.35E+05
132	630	3.95E-02	818	2.09E-07	6.85E+05
132	800	3.24E-02	888	2.17E-07	7.95E+05
132	1000	2.75E-02	949	2.38E-07	8.60E+05
220	500	4.89E-02	732	1.39E-07	8.15E+05
220	630	3.91E-02	808	1.51E-07	8.50E+05
220	800	3.19E-02	879	1.63E-07	9.75E+05
220	1000	2.70E-02	942	1.77E-07	1.00E+06
400	800	3.14E-02	870	1.30E-07	1.40E+06
400	1000	2.65E-02	932	1.40E-07	1.55E+06
400	1200	2.21E-02	986	1.70E-07	1.70E+06
400	1400	1.89E-02	1015	1.80E-07	1.85E+06
400	1600	1.66E-02	1036	1.90E-07	2.00E+06
400	2000	1.32E-02	1078	2.00E-07	2.15E+06

Table S4. HVDC cable parameters considered in the analysis (18).

Voltage (kV)	Size (mm^2)	Resistance (Ω/km)	Current (A)	Cost (GBP/km)
150	1000	2.24E-02	1644	6.70E+05
150	1200	1.92E-02	1791	7.30E+05
150	1400	1.65E-02	1962	7.85E+05
150	1600	1.44E-02	2123	8.40E+05
150	2000	1.15E-02	2407	9.00E+05
220	1000	2.24E-02	1644	8.55E+05
220	1200	1.92E-02	1791	9.40E+05
220	1400	1.65E-02	1962	1.02E+06
220	1600	1.44E-02	2123	1.09E+06
220	2000	1.15E-02	2407	1.18E+06

A power-to-hydrogen (PtH) system is considered as a potential candidate to forgo cables, the installation time and effort, the area requirement, fixed infrastructure (and accompanying opposition), regulatory requirements associated with leasing, installing, and permitting associated with cable transmission systems. In this system, the electricity generated at the turbines is collected at a single collector platform using inter-array cables between turbines and the platform where an OfSS is located, similar to the HVDC system. At the OfSS the electricity is used in an electrolyzer in which desalinized water is electrolyzed into hydrogen and compressed for shipping to shore. A hydrogen tanker is used to periodically collect the hydrogen generated and stored at the collector site to a storage facility on shore (19, 20). One potential benefit of this system is the flexibility in delivery that it can accommodate. A single hydrogen ship may collect hydrogen from several locations (OSW plants) and deliver the hydrogen to several locations on shore (different storage facilities).

Note 3. Distribution of Normalized Metrics

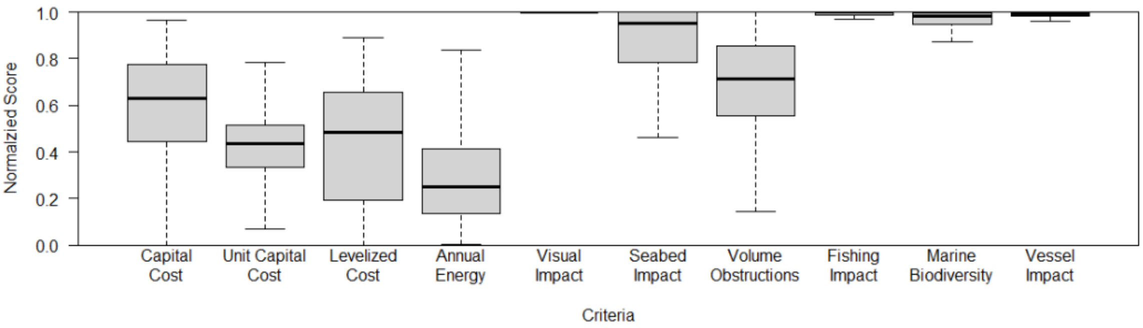

Figure S3. Distribution of normalized metrics across the entire alternatives space.

Note 4. DP Thruster Consumption

Following the method in Santarromana et al. (21) we compute the thruster consumption in a three-step process:

1. Estimate the environmental thrust forces under a range of conditions for each turbine size.
2. Considering several DP Thruster configurations (numbers of thrusters on the turbine), calculate the expected annual thruster consumption.
3. Calculate the levelized cost of energy (LCOE) from the thruster investment for each of the DP thruster configurations.
4. The minimum LCOE configuration is optimal for the turbine, and the resulting consumption from the configuration is used.
5. Extrapolate the results across several mean wind speeds.

Step 1: Environmental thrust forces

Wind, wave, and current forces are considered using a design load case (DLC) by the American Bureau of Shipping (ABS) for station-keeping design for floating OSW turbines (22). The parameters used are shown in Table S5.

Table S5. Environmental loading conditions, parameters used, and orientations to compute the total environmental thrust force on the turbines.

Parameter	Conditions Used
Design Load Case (22)	DLC 1.3
Wind Speed (at hub height), V_{hub}	$V_{\text{cut-in}} < V_{\text{hub}} < V_{\text{cut-out}}$
Surface Current Speed, V_{curr}	The wind-generated currents, $V_{\text{curr}} = \mathbb{E}[V_{\text{curr}} \vert \mu(V_{\text{hub}})]$
Wave Height, H	Normal Sea State, $H = \mathbb{E}[H \vert V_{\text{hub}}]$
Wave Period, T	(Not defined)
Wind and Wave Directionality	Unidirectional wind and wave directions

The total thrust force, $\vec{F}_{\text{tot}}$, is the vector combination of the wind, wave, and current force vectors,

$$\vec{F}_{\text{tot}}(V_{\text{hub}}) = \vec{F}_{\text{wind}}(V_{\text{hub}}) + \vec{F}_{\text{curr}}(V_{\text{curr}}(V_{\text{hub}})) + \max_{H,T}\left(\vec{F}_{\text{wave}}(\hat{H}, \hat{T})\right) \tag{S1}$$

Step 2: Expected annual thruster consumption

ABS provides a generalized relationship between input power and applied force for the DP thrusters in equation (S2) (22, 23). Equation (S3) gives the power as a function of applied force for a single thruster. We assume the force is applied equally across all thrusters and each consumes an equivalent amount of power. The power consumption of a system consisting of n thrusters is estimated using equation (S4).

$$F_1 = K(P_1 D)^{\frac{2}{3}} \tag{S2}$$

$$P_1 = \frac{1}{D}\left(\frac{F_1}{K}\right)^{\frac{3}{2}} \tag{S3}$$

$$P_{\text{DP}}(F_{\text{tot}}, n) = \frac{n}{D}\left(\frac{F_{\text{tot}}}{n\,K}\right)^{\frac{3}{2}} \tag{S4}$$

where F_1 is the bollard pull force (N), P_1 is the power required (kW) for a single thruster, K is a constant equal to 1,250, and D is propeller diameter (m) (23). F_{tot} is the needed station-keeping force (equation (S1)), and P_{DP} is the DP system power requirement (kW).

The annual expected thruster energy consumption, $\mathbb{E}_{\mathrm{TC}}$, is computed as an expectation of the power consumption of the DP system considering a probability density function of wind speeds at hub height, $f(V_{\mathrm{hub}})$—for which we consider a Weibull distribution.

$$\mathbb{E}_{\mathrm{TC}} = 8760\left[\int_{V_{\mathrm{hub}}} f(V_{\mathrm{hub}})\ P_{\mathrm{DP}}(F_{\mathrm{tot}}(V_{\mathrm{hub}}), n)\ dV_{\mathrm{hub}}\right] \quad \text{(S5)}$$

The expected wind turbine generation, $\mathbb{E}_{\mathrm{GEN}}$, is computed similarly, with the wind power curve in place of the thruster power consumption function in equation (S5).

We compute this expected energy consumption under two wind speed classes adopted from the National Renewable Energy Laboratory (NREL) Annual Technology Baseline (ATB) for OSW (24). Along with different wind speed probability distributions, the wind speed classes are also characterized by different plant deployment, operation, financing, and operations & maintenance costs that we use to calculate the cost effectiveness of the systems in the next step.

Step 3: Compute LCOE for the DP turbine configurations

The calculation method employed by the NREL ATB to calculate the LCOE of the turbine is used (24):

$$\mathrm{LCOE} = ((\mathrm{CRF} \times \mathrm{PFF} \times \mathrm{CFF} \times \mathrm{netOCC}) + \mathrm{FOM}) \times 1000/(\mathrm{netCF} \times 8760)$$

where CRF, PFF, and CFF are the capital recovery, production finance, and construction finance factors, respectively. The net CF for the turbine is calculated as,

$$\mathrm{netCF}_{\mathrm{turbine}} = \frac{\mathbb{E}_{\mathrm{GEN}} - \mathbb{E}_{\mathrm{TC}}}{8760 \times \mathrm{turbine\ size}}$$

The net overnight capital cost, netOCC, for DP is adjusted from the NREL base overnight capital cost by reducing it by the proportion estimated for mooring and increasing it by the DP system cost ($/kW).

Step 4: Identify the LCOE-minimizing configuration

Once the LCOE is computed for all the configurations of DP thruster systems, and for each turbine, the configuration that minimizes the LCOE is the configuration required for the turbine. The results are reported in Table S6. The thruster consumption percent (TCP) is also reported under the three wind speed distributions (described by their annual mean wind speed). The TCP is computed as,

$$\mathrm{TCP} = \frac{\mathbb{E}_{\mathrm{TC}}}{\mathbb{E}_{\mathrm{GEN}}}$$

And this parameter gives the percent of annual generation that is consumed by the thrusters. In areas where the annual mean wind speed is different, the TCP may be different, as evidenced by the differences in the TCP when computed for different wind speed classes.

Table S6. Number of thrusters for the LCOE-minimizing DP configuration, and the TCP under different wind profiles, with different wind probability distribution functions (defined using only the mean).

Turbine Rated Power [MW]	5	8	10	15
LCOE minimizing configuration (number of 5 m thrusters)	4	4	5	13
TCP (annual mean of the wind profile = 7.1 m/s)	39%	30%	38%	72%
TCP (annual mean of the wind profile = 8.9 m/s)	32%	23%	32%	64%
TCP (annual mean of the wind profile = 9.6 m/s)	30%	21%	30%	61%

<u>Step 5: Extrapolate the results across different annual mean wind speeds</u>

Using the results of TCP given wind speed profiles in Table S6, a linear model for each turbine size that relates mean windspeed to the TCP is calculated (see Figure S4). The TCP gives the expected proportion of annual generated energy that is consumed by the thrusters, and therefore, turbines that employ DP have an annual electricity output that is reduced by the TCP.

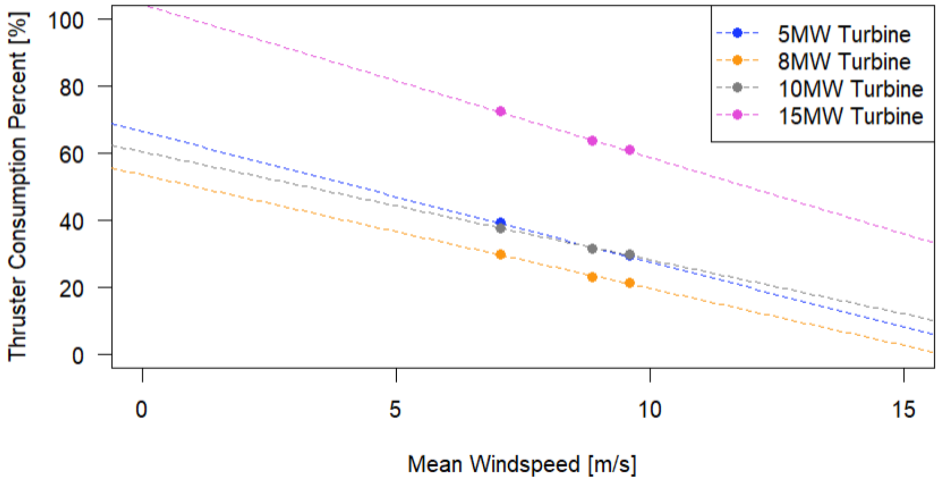

Figure S4. Linear models to compute TCP for each turbine size at various annual mean wind speeds.

Using this linear model, given a turbine size and a mean wind speed for a given grid square, the TCP is calculated and the expected net energy generation by a turbine in the grid square is calculated as,

$$\mathbb{E}_{\text{DP turbine}}\left[\frac{\text{MWh}}{\text{year}}\right] = \mathbb{E}_{\text{turbine}} \times \left(1 - \text{TCP}(V_{\text{wind}}, T)\right)$$

where the expected annual energy generation of a DP turbine, $\mathbb{E}_{\text{DP turbine}}$, is the equal to that of a non-DP turbine, $\mathbb{E}_{\text{turbine}}$, reduced by the TCP.

Note 5. Turbine Asset Cost and Efficiency Computations

The cost of the system is quantified as the overnight capital cost of the system which considers the procurement cost of the turbine, substructure (monopiles are considered for fixed-bottom, and

semisubmersibles are considered for floating foundations), inter-array cables, onshore spur line to interconnect to the existing transmission system, station-keeping system, and the export system. Turbine costs come from (25) and vary with turbine rated capacity, monopile costs come from (26) and vary with water depth, semisubmersible costs come from (15) (sized for a 15 MW turbine, the same is used for all turbines).

Mooring costs come from (27) assuming three catenary mooring chains. DP costs are computed considering the number of thrusters needed (see Table S6). Inter-array cable costs use unit costs from (28), and assume the grid spacing of 2 km. Onshore spur line cost is estimated assuming $2 Million/mile of distance from landfall to a point of interconnection (29).

A description of the cost assumptions used for the components of each value chain is given in Table S7. The costs of are calculated given the physical parameters (distance, depth, wind speed, etc.) and components of the value chain.

Table S7. Cost assumptions and sources for value chain components.

Parameter	Value	Source
**TURBINES**		
Turbine cost	1,300 USD/kW	Beiter et al. (25)
Turbine annual O&M	2% of CAPEX/year	Van Wingerden et al. (30)
**SUBSTRUCTURES**		
Steel cost	1,000 USD/ton	Myhr et al. (26)
Monopile steel consumption	40 ton/m of water depth	Myhr et al. (26)
Semisubmersible steel consumption	3,900 ton/substructure	Allen et al. (15)
Monopile annual O&M	2% of CAPEX/year [b]	Van Wingerden et al. (30)
Semisubmersible annual O&M	2% of CAPEX/year [b]	Van Wingerden et al. (30)
**STATION KEEPING**		
Mooring chain breaking load (MBL)	4500 kN	(31)
Mooring chain cost	0.0591*MBL-87.6 USD/m	Beiter et al. (27)
Mooring annual O&M	1% of CAPEX/year [c]	Avanessova et al. (32)
Drag embedment anchor cost	10.198*MBL USD/anchor	Beiter et al. (27)
DP thruster cost	1,700,000 USD/thruster	(33)
DP annual O&M	4% of CAPEX/year	(34)
**ARRAY CABLES**		
Cable cost	45,000 USD/km	Jung et al. (28)
Array cable annual O&M	3% of CAPEX/year	Van Wingerden et al. (30)
Array cable length	2 km/turbine	U.S. Coast Guard (35)
**EXPORT SYSTEM**		
Collector platform cost	6,710,000 USD/platform	Xiang et al. (36)
HVDC terminal fixed cost [a]	30,750,000 USD	Xiang et al. (18)
HVDC terminal variable cost [a]	134,070 USD/MVA	Xiang et al. (18)
HVDC export cable cost	(see Table S4) [a]	Xiang et al. (18)
HVDC system annual O&M	3% of CAPEX/year	Van Wingerden et al. (30)
HVAC terminal fixed cost	6,150,000 USD	Xiang et al. (18)
HVAC terminal variable cost	55,350 USD/MVA	Van Wingerden et al. (30)
HVAC export cable cost	(see Table S3)	Van Wingerden et al. (30)
HVAC system annual O&M	2.5 % of CAPEX/year	Van Wingerden et al. (30)
Electrolyzer cost	900 USD/kW	Saba et al. (37), median for projection years beyond 2010
Electrolyzer annual O&M	1.5% of CAPEX/year	Van Wingerden et al. (30)
Transformer cost	1,300,000 USD/MW	Xiang et al. (36)
Transformer annual O&M	2.3% of CAPEX/year	Van Wingerden et al. (30)
Hydrogen shipping vessel cost	440,000,000 USD/ship	Alkhaledi et al. (19)
Hydrogen ship annual O&M	4% of CAPEX/year	Alkhaledi et al. (19)
Hydrogen production rate	67 kWh/kgH_2	IRENA (38)
Hydrogen loss in liquefication	15%	Popov & Baldynov (39)
**ONSHORE CABLE**		
Onshore spur line	2,000,000 USD/mile	Saadi et al. (29)
Onshore cable annual O&M	3% of CAPEX/year	Van Wingerden et al. (30)

[a] The number of cables needed for the export cable system considers the physical capabilities of the cable options, and therefore is calculated for a set of cables and functions from (18, 36).
[b] Assumed to be the same as the turbine annual O&M percent.
[c] The failure rate of a single chain times number of chains.

Comparing Cost Model. We compare the cost model developed in this analysis to an analysis of OSW sites done by the National Renewable Energy Laboratory (NREL) (25). Three OSW sites are assessed for their costs, potential energy generation, and LCOE. Table S8 summarizes the results from the NREL model and the results attained from this analysis for each of the three sites.

Table S8. Comparative costs calculated using NREL models (25) and the model used in this analysis. Major differences in the costs computed are highlighted.

	Morro Bay		Diablo Canyon		Humboldt	
	**NREL**	**Our Model**	**NREL**	**Our Model**	**NREL**	**Our Model**
Turbine ($/kW)	1,297	1,300	1,297	1,300	1,297	1,300
Substructure ($/kW)	1,235	1,170	1,235	1,170	1,235	1,170
Array Cables	291	90	258	90	275	90
Export Cables	454	498	487	484	447	410
Onshore spur line	78	65	78	17	78	13
**Total Electric System ($/kW)**	823	653	823	591	800	513
Installation Cost ($/kW)	333	0	306	0	269	0
Development	142	0	141	0	139	0
Lease Price	88	0	88	0	88	0
Project Management	74	0	73	0	72	0
Insurance	46	0	46	0	45	0
Project Completion	46	0	46	0	45	0
Decommissioning	50	0	45	0	39	0
Procurement Contingency	213	0	213	0	211	0
Install Contingency	100	0	90	0	78	0
Construction Financing	191	0	189	0	186	0
**"Soft" CAPEX ($/kW)**	950	0	931	0	903	0
**Total CAPEX ($/kW)**	4,638	[redacted]	4,592	[redacted]	4,504	[redacted]
**Total CAPEX of Included Items ($/kW)**	3,355	3,123	3,355	3,061	3,332	2,983
**Plant Characteristics**						
LCOE (USD/MWh)	90	100	92	102	81	80
Capacity Factor	49%	38%	48%	39%	53%	45%
**Site Characteristics**						
Average Shore Distance (km)	43-53		43-67		36-46	
Average Water Depth (m)	830-1067		495-840		608-890	
Average Port Distance (km)	192-235		206-232		253-292	

As Table S8 demonstrates, the main differences between the costs calculated in this model compared with the more detailed NREL model are the inclusion of installation costs and project execution costs in the latter. The costs for plant components are closely represented aside from the array cable costs, which the model in this analysis underestimates compared to the NREL calculations. Furthermore, the capacity factor for the plant sites is underestimated by the model in this analysis, and as a result, the LCOE is overestimated by this model—both conservative estimates for these parameters, and thus this model is conservative in its outputs.

As there is discrepancy between the sophisticated NREL model and this analysis, we compare the relative differences of capital costs and LCOE (two of the criteria in the MCDM) in Table S9. The MCDM in this analysis compares relative, normalized values (as opposed to absolute values), and Table S9 shows that these relative differences of these criteria calculated using the NREL model are closely captured with the model in this analysis.

Table S9. Indexed values for capital costs and LCOE between an NREL model (25) and the models used in this analysis illustrating how the relative differences compare between the models.

Relative Differences (Indexed to Diablo Canyon)	Morro Bay	Diablo Canyon	Humboldt
**CAPEX Overall - NREL**	101	100	98
**CAPEX - Our Model**	102	100	97
**LCOE - NREL**	98	100	88
**LCOE - Our Model**	98	100	78

Note 6. Novel Deployment Options

Table S10. A description of the novel deployment options and the benefits and costs realized by these options.

Deployment Option	Description	Benefits	Costs
Baseline Facility	• 10 MW Turbine<br>• Moored Foundations<br>• HV Cable Transmission<br>• 1,000 MW Plant	• Accepted method for next generation deep water deployment	• Cost sensitive to water depth<br>• Depth is a limitation<br>• Cost sensitive to shore distance
Novel Deployment Facility	• 10 MW Turbine<br>• DP Foundations<br>• PtH Transmission Pathway<br>• 1,000 MW Plant	• Eliminate cost sensitivity to shore distance<br>• Eliminate depth limit (more area is available for deployment)<br>• Eliminate disrupted seabed from cables<br>• Eliminate ocean obstructions from mooring chains	• Highest capital cost deployment option<br>• Reduced energy output (DP consumption)<br>• Reduced energy efficiency (Hydrogen conversion efficiency)

Note 7. Additional Results – Distribution of Normalized Scores based on Plant Scale

Figure S5 illustrates the distribution of each normalized metric that contributes to the suitability scores for the smallest (200 MW) and largest (1,400 MW) plant scales considered in this analysis.

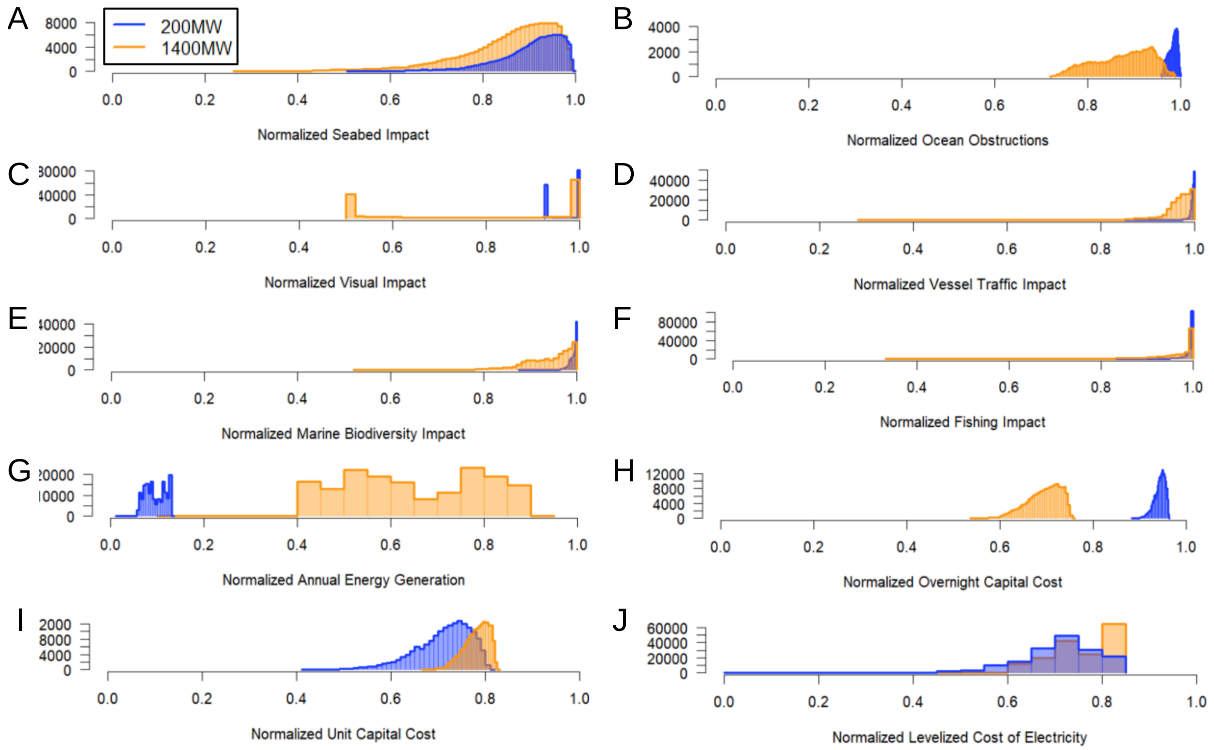

Figure S5. A) – J) Distribution of all 10 normalized metrics for the smallest and largest plant sizes considered in this study; each observation in the histograms are site locations for the plant. Normalized values reflect whether the metric is a cost or a benefit already, therefore, a value of one indicates the preferred outcome in each.

The stakeholder suitability score is defined by the scores in Figure S5-A to Figure S5-G. The implication of this is that small capacity plants have less variance in stakeholder perspectives across potential sites. While the outlay of energy output from larger plants dominates that of smaller plants (Figure S5-G), a smaller physical presence results in a tight distribution of normalized scores for the physical impact metrics of the plant (Figure S5-A to Figure S5-F). The developer suitability score is defined by the scores in Figure S5-G to Figure S5-J. The smaller plant realizes a wider distribution of unit capital cost and LCOE, while a tighter distribution of overall capital cost is seen.

Note 8. Additional Results – Other Weight Profiles

Figure S6 illustrates the suitability score maps of the baseline value chains under uniform-weighting. These results can be compared to the stakeholder and developer weighting maps in Figure 2 of the main text. The stakeholder-weighted and uniform-weighted maps appear very similar. As Dawes has noted, uniformly weighting criteria is good policy, as it requires fewer assumptions, and can suitably represent expert judgements (40, 41). This suggests that stakeholder-weighted maps should be used to a greater extent in matters regarding OSW project decision-making.

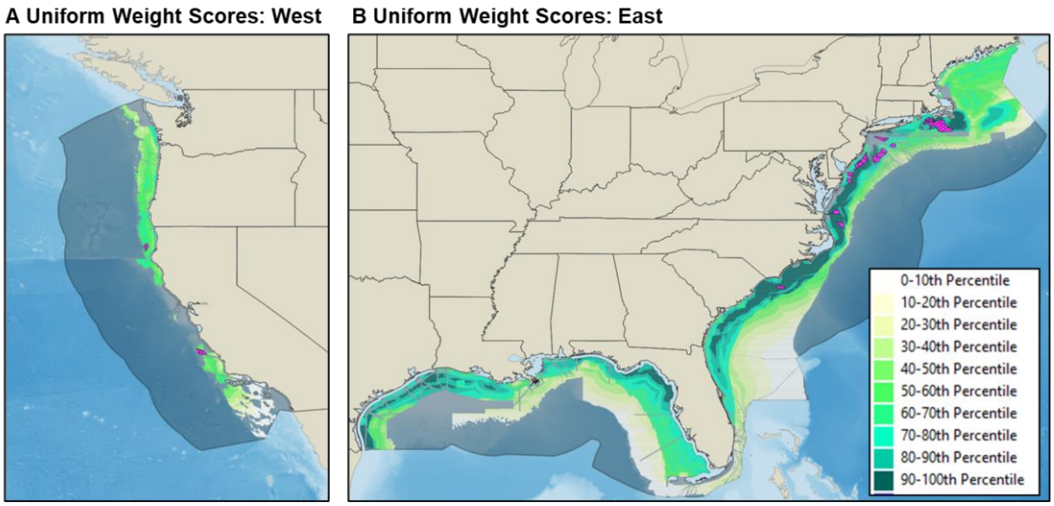

Figure S6. Maps of the baseline value chain scores for the baseline value chains under uniform weighting.

Figure S7 illustrates the distribution of scores with varying plant scale of all the weight profiles. These are demonstrated for the baseline facility with different plant capacities.

A – **Low Concern (energy output lower weight than other impacts)**

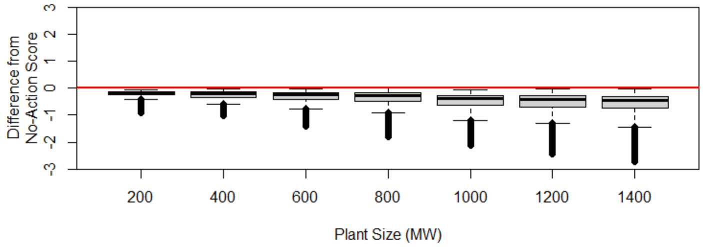

B – **Stakeholder Weights (energy output equally weighted with other impacts)**

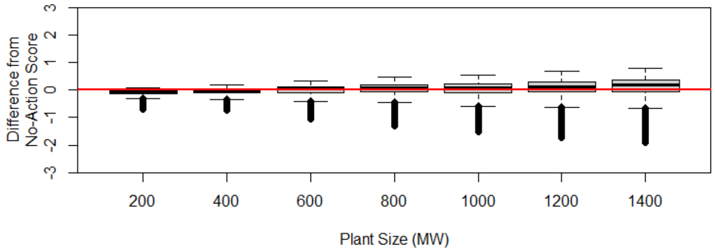

C – **High Concern (energy output higher weight than other impacts)**

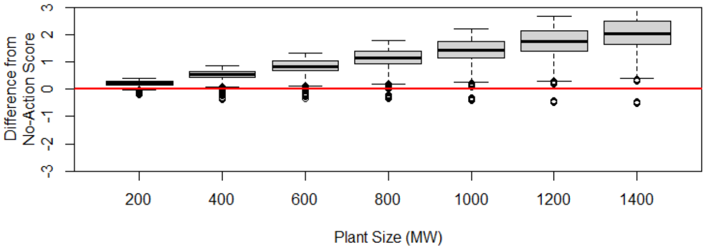

D – **Uniform Weights (equivalent weights across all metrics)**

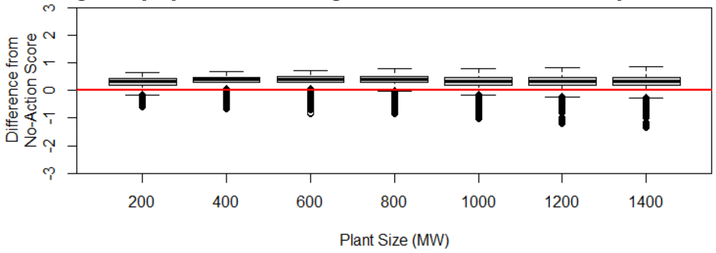

E – **Developer Weights (equivalent weight across cost and output metrics only)**

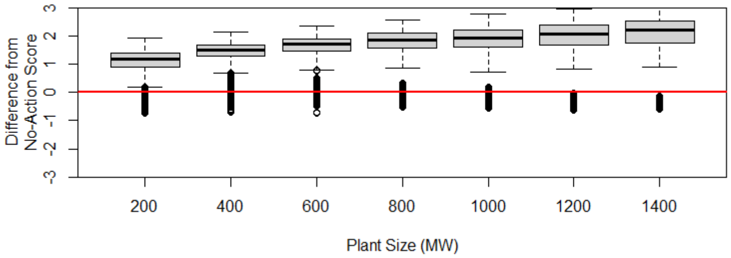

Figure S7. The distribution of suitability scores by plant size considering 10 MW fixed foundation turbines, and DC cable transmission under various weighting paradigms (see Table S19).

As concern for deploying more clean energy increases relative to other impacts, the suitability of all plants increases, but larger plants increase by even more. When there is high concern for deploying clean energy (Figure S7-C) nearly all site alternatives for the largest plant dominate all

the small plant site alternatives. When concerns for deploying more clean energy are low, small are preferred to large plants. Smaller plants are tightly distributed, and achieve similar scores regardless of the level of concern for energy output (Figure S7-A to Figure S7-C), and therefore, smaller plants are more robust to the institutional context if there is considerable uncertainty of the context the plant is being deployed in.

The context in which a renewable energy plant is deployed will play a role in the potential success of the plant. Furthermore, while we have reported on a national-scale model, perspectives may change at more granular scales, from community to community. As demonstrated here, there are certain options that are more robust (scores do not vary greatly) to different institutional contexts (represented by different weight profiles).

Our investigation of the effects of scale on suitability adds to the conversation surrounding the implications of project scale. Recent work suggests that smaller, granular deployments and technologies which do not rely on intensive customization are more conducive to faster growth and can thus be deployed more quickly in pursuit of urgent decarbonization (42–44). Preference has also been exhibited for smaller generation plants among local communities in southern Italy (45) and Germany (46) through choice experiments for terrestrial wind, mainly due to aesthetic reasons. Our results show that smaller plants exhibit a tight distribution of cumulative stakeholder impacts, demonstrating robustness to plant location. Regarding the techno-economic implications of scale, smaller plant sizes have better cost control as evidenced by a tighter distribution of capital costs (see Figure S5-H). There are also cost discontinuities with increasing scale arising from discrete component capacities and installation windows that impact the overall project timeline. Our study considered discrete cable capacities (see Table S3 and Table S4) and turbine sizes (see Table S1). While we considered plant alternatives to have a single collector site due to our site aggregation method, real-world plants may have more when plant capacities are larger. Furthermore, plant commissioning time takes several years (47) and increases discontinuously because installation is constrained to a few months of the year. Taken together, these contribute to a discontinuous LCOE as plant size increases (with all other aspects constant). In this study, we considered overnight costs to estimate LCOE, and therefore consider the discontinuities arising from discrete component capacities, while the temporal impact of scale—reducing the plant size reduces the overall installation time and costs—is an interesting effect to consider in future work.

Project development typically occurs in stages: sites are identified based on a set of criteria; design and development then begin, and so does stakeholder engagement. However, decisions regarding site, scale, and technology should not be made independently. As Figure 3 of the main text illustrates, we find that areas that are suitable for some plant scales are not suitable for others. Project developers looking to deploy plants of a certain scale should be mindful of this when planning their project. Our study is the first to incorporate the impact on suitability of different OSW plant sizes, owing to our turbine site aggregation method. Furthermore, an analysis of a granular set of alternatives and sites as done here should be preferred to project-by-project stakeholder impact assessments. The former gives a fuller view of where cumulative impacts are the greatest, while the latter may result in developments being placed where the ability for stakeholders to petition decision-makers is weaker, regardless of how the cumulative impacts compare to other areas.

Our results suggest that the poor track record of deploying U.S. OSW projects is unsurprising. Prior (and some current) site and design decisions align with the developer paradigm but not with the stakeholder one, offering an explanation for failed deployments. While all early projects have been sited close to shore (Table S13) tending to optimize techno-economic criteria, we demonstrate that projects can move, even marginally, further from shore and remain within consensus areas (Figure 4 of the main text) balancing techno-economic and plant impact criteria. The implications for developers and policy makers are threefold. First, developers that have a strong preference for large projects should limit their attention to locations with higher scores under the stakeholder paradigm. To avoid failed deployment, policy makers should develop

strategies that incentivize such choices. Second, while smaller plants yield higher unit and levelized costs, they might prove appropriate in more locations and institutional contexts than larger plants. Third, to facilitate development along the U.S. West Coast and other coasts where depths increase rapidly, policy makers should provide research support and financial incentives to increase the viability of novel technologies that are deployed far offshore and score highly under the stakeholder paradigm.

Note 9. Robustness Checks

Table S11 provides a qualitative analysis of the categories of metrics that may be included in the developer and stakeholder weight profiles. In the first column, whether the metric is a benefit or a cost is described. Given that the No-Action choice realizes a metric of zero—as the plant alternative is not built—when it is normalized, the No-Action score would not change if the metric is a benefit, and the No-Action score would increase by one if the metric is a cost when the metric is part of the weight profile.

For each metric type (benefit or costs) there are four combinations of inclusion or exclusion in the stakeholder and developer weight profiles, and the impact for each category of metrics on the stakeholder and developer score difference to the No-Action is then reported in Table S11. For example, taking the first row of the table, including a benefit metric that will end up being part of both the stakeholder and developer weight profiles will not change the No-Action score, but plant alternatives will have a metric value for the metric that is greater than or equal to zero. After normalizing the metric, it would elevate the stakeholder and developer scores for plant alternatives by a value between zero and one times the applied weight on this metric. An example of a metric that fits this category is job creation—it is likely seen as a benefit for both developers and external stakeholders. No-Action creates no jobs, and therefore does not change the No-Action score, while plant alternatives would create jobs and therefore increase the difference between plant alternative scores and the No-Action score by a value between one and zero times the applied weight on the criterion. Examples of metrics are provided in Table S11, including how these metrics might already be related to the datasets included in the study.

Table S11. Categories of metrics and how their inclusion might impact the main results.

Type of metric (Change to No-Action Score if included)	Stakeholder weight profile inclusion	Developer Weight Profile inclusion	Impact on Stakeholder Score Difference	Impact on Developer Score Difference	Examples (likely relation to included datasets)	Impacts on Main Numerical Suitability Score Results
Benefit (+ 0)	Yes	Yes	+ [0, w]	+ [0, w]	• Job creation (likely related to plant size)<br>• Pollution reduction (likely related to annual energy output)	Elevate all scores, decrease estimate of “at-risk” plant proposals, increase amount of consensus areas
Benefit (+ 0)	Yes	No	+ [0, w]	0		Elevate Stakeholder scores, decrease estimate of “at-risk” plant proposals, increase amount of consensus areas
Benefit (+ 0)	No	Yes	0	+ [0, w]		Elevate Developer scores
Benefit (+ 0)	No	No	0	0		No effect
Cost (+ w)	Yes	Yes	- [0, w]	- [0, w]	• Construction impacts (likely related to plant size)<br>• Habitat loss (likely related to biodiversity dataset)<br>• Noise (likely related to plant size and type of substructure)<br>• Military operations (related to vessel presence dataset)	Depress all scores, increase estimate of “at-risk” plant proposals, decrease amount of consensus areas
Cost (+ w)	Yes	No	- [0, w]	0		Depress Stakeholder scores, increase estimate of “at-risk” plant proposals, decrease amount of consensus areas
Cost (+ w)	No	Yes	0	- [0, w]		Depress Developer scores
Cost (+ w)	No	No	0	0		No effect

There are three main numerical results given in the study: the distribution of stakeholder and developer scores for the entire alternatives space, the scores of US plant proposals (and “at-risk” proposals), and the amount of consensus areas on each coast. The final column of Table S11 provides a qualitative description of how each category of metrics might impact these main results. Among potential metrics, there are likely more costs than benefits that are missing from the analysis as certain environmental and industry impacts can be further disaggregated—this is demonstrated by the number of possible examples of missing metrics in the table for which there are more possible cost metrics missing than benefit metrics. The result of including more cost metrics (or adding more cost metrics than benefit metrics in a future iteration of this work) would be that the estimate of ‘at-risk’ capacity increases, and the amount of consensus areas decreases. Therefore, the study presented here may give a more optimistic view of suitability of plant alternatives.

There are two more qualitative or conceptual results from this study: the spatial distribution and behaviors of the weight profiles, and the variance of small plant scores vs large plant scores. Both of these conceptual results should not be fundamentally changed by the inclusion of other metrics. Regarding the impact of plant scale on the variance of suitability scores, the inclusion of any missing metrics should not affect the main outcome—that smaller plants are more robust (with a tighter variance of scores) than larger plants. Regarding the behavior with distance to shore, none of the missing metrics should counteract the outcome that stakeholder scores tend to be higher away from shore while developer scores tend to be higher closer to shore. Either the metrics would be distributed independent of distance to shore (as with job creation or pollution reduction which likely is more related to plant size and output), or the main effects realized from the current study would be enhanced. For example, habitat loss and military operations are likely

related to the biodiversity and vessel presence datasets already included in the analysis, and these would disfavor nearshore alternatives. As these two cost metrics would likely be part of the stakeholder weight profile, the effect realized in the current study—that near shore alternatives score lower from a stakeholder perspective—would therefore be enhanced. Therefore, these results are robust to missing metrics.

Because many of the identified missing metrics are likely related to existing metrics in the current study, we conduct a more comprehensive sensitivity analysis on each metric. As some metrics may be derived from the existing datasets, including them in the study would be operationally similar to having an increased weight on the metrics they are related to. We therefore conduct a sensitivity analysis where each of the 10 metrics are included but provide a disproportionately large weight on one of the metrics, resulting in 10 new weight profiles. For each of the 10 additional weight profiles, one metric is given a weight that is more than 10 times larger than the others, while the remaining nine metrics have an equivalent weight. These additional weight profiles are given in Table S12.

Table S12. Additional 10 single-metric-skewed weight profiles considered.

**Metric**	$Z_{NA,j}$ [a]	**Weight profiles skewed toward one metric**									
Overnight Capital Cost	1	**5.5**	0.5	0.5	0.5	0.5	0.5	0.5	0.5	0.5	0.5
Unit Overnight Capital Cost	1	0.5	**5.5**	0.5	0.5	0.5	0.5	0.5	0.5	0.5	0.5
Annual Energy/H_2 Output	0	0.5	0.5	**5.5**	0.5	0.5	0.5	0.5	0.5	0.5	0.5
LCOE/LCOH	0	0.5	0.5	0.5	**5.5**	0.5	0.5	0.5	0.5	0.5	0.5
Visual Impact	1	0.5	0.5	0.5	0.5	**5.5**	0.5	0.5	0.5	0.5	0.5
Impact on Fishing	1	0.5	0.5	0.5	0.5	0.5	**5.5**	0.5	0.5	0.5	0.5
Impact on Marine Life	1	0.5	0.5	0.5	0.5	0.5	0.5	**5.5**	0.5	0.5	0.5
Impact on Vessel Traffic	1	0.5	0.5	0.5	0.5	0.5	0.5	0.5	**5.5**	0.5	0.5
Disrupted Seabed	1	0.5	0.5	0.5	0.5	0.5	0.5	0.5	0.5	**5.5**	0.5
Obstructions	1	0.5	0.5	0.5	0.5	0.5	0.5	0.5	0.5	0.5	**5.5**
**No-Action Score** [b]	–	**9.0**	**9.0**	**4.0**	**4.0**	**9.0**	**9.0**	**9.0**	**9.0**	**9.0**	**9.0**

[a] The normalized No-Action metrics computed as defined in equation (2) of the main text.
[b] Maximum of 10 possible, computed as described in equation (6) of the main text.

The results of the sensitivity analysis are shown in Figure S8. As seen with the results, the variance of the smaller plants (provided in the figure) is smaller than that of large plants for each of the weight profiles except the unit overnight capital cost skewed, cost of energy skewed, and the disrupted seabed skewed weight profiles—in each of these, the smallest plant does not exhibit the smallest variance. If weight profiles were heavily skewed toward these three metrics, we might see the opposite effect than what was found in the main results and see that small plants have greater variance of scores than large plants. This might occur for a heavily techno-economic optimizing decision-maker, for example. The remaining single-metric-skewed weight profiles exhibit a tighter variance for smaller plants than larger ones as found with the main results, and therefore, that result would hold when a disproportionate amount of weight is placed on these metrics.

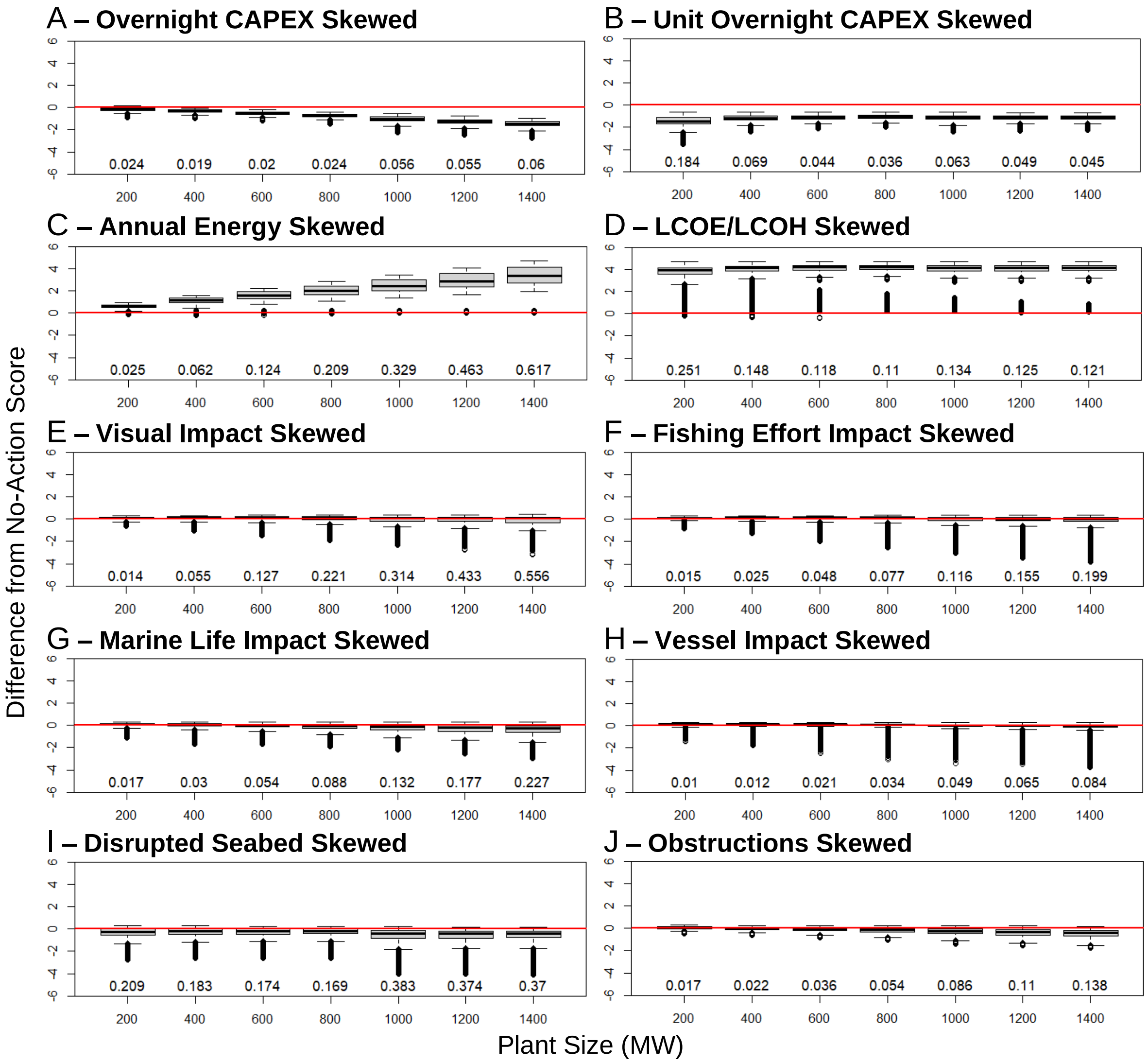

Figure S8. Effect of single-metric-skewed weights on suitability scores of alternatives. Shown by plant size as the difference from the No-Action score of that weight profile. Numbers below each boxplot give the variance of the suitability scores for alternatives of that plant size.

Note 10. Assessing other plant proposals

Proposals follow the developer paradigm, but scores are depressed under the stakeholder paradigm

We calculate suitability scores under both developer and stakeholder paradigms for several existing and past OSW project proposals in the U.S., listed in Table S13. While this is not intended to be a predictive model, the scores give insight as to the potential suitability of these proposals. Further site-specific assessments are needed beyond the suitability scores we provide here. The projects included in the table are those that have a construction and operations plan (COP) submitted to the U.S. Department of the Interior, signifying their advanced stage in the development process, and with project parameters defined (plant size and turbine size being considered)—projects with a lease area, but no COP available (as of March 2024) are not

included. The project envelope is typically described in the COP, which is why we only include projects that have submitted one, as the project boundaries are well-defined at the point of COP submission. Expectedly, all projects score highly under the developer paradigm, illustrating its usefulness at approximating developer decision-making. However, under the stakeholder paradigm, the scores are depressed, though several remain positive, if only marginally. The cancelled Cape Wind project experienced stakeholder challenges (48), and scores negatively under our stakeholder paradigm. Vineyard Wind, another project to face litigation and scrutiny from external stakeholders, scores marginally positive under the stakeholder paradigm (49). Of the proposed capacity listed in the table, nearly 2 GW—not including two cancelled projects, Cape Wind and Ocean Wind 1—have low stakeholder suitability scores of + 0.1 or less (setting the Vineyard Wind score as the threshold as it also experienced stakeholder resistance), indicating they may face stakeholder risk. Interestingly, while the cancelled Ocean Wind 1 cites macroeconomic factors as the reason for its cancellation and not stakeholder challenges, it still realizes a low stakeholder suitability score.

Table S13. Existing and past U.S. project proposals in advanced development. Note: all proposals consider high voltage export cables and fixed foundations with monopiles in their proposals. A negative score indicates that the No-Action suitability score is greater than the alternative's suitability score. Distances reported are the straight-line distance to the nearest point to shore from the centroid of the lease area; actual cable distances may be longer depending on the chosen cable landfall site.

Proposed OSW project	Lease Area	Shore dist (km)	Plant size (MW)	Turbine Size (MW)	Stakeholder score [a]	Developer score [a]
Cape Wind[x] (48)	Not Specified	14	468	5	– 1.0 to – 0.7[g]	+ 2.0 to + 2.2[g]
Maryland Offshore Wind (50)	OCS-A 0490	26	2,000 [b]	≤18 [b]	+ 0.3	+ 3.0
Atlantic Shores North (51)	OCS-A 0549	29	2,355 [b]	12-20 [c]	+ 0.2	+ 2.9
Ocean Wind 1[d x] (52)	OCS-A 0498	30	1,100	12	+ 0.1 to + 0.3	+2.5 to + 2.7
Atlantic Shores South (53)	OCS-A 0499	30	1,510	12-20 [c]	+ 0.3	+ 3.0
Revolution Wind (54)	OCS-A 0486	36	704-880	8-12	0.0 to + 0.2	+ 2.1 to + 2.4
Empire Wind (55)	OCS-A 0512	39	2,200 [b]	15 [c]	+ 0.3	+ 2.8
South Fork Wind (56)	OCS-A 0517	42	90-180 [b c]	6-12	0.0	+ 1.5 to + 1.6
Vineyard Wind (57)	OCS-A 0501	55	800	12	+ 0.1 to + 0.3	+ 2.2 to + 2.3
Sunrise Wind (58)	OCS-A 0487	55	924-1,034	11	+ 0.3	+ 2.2 to + 2.3
Coastal Virginia Offshore Wind (59)	OCS-A 0483	57	2,800 [b]	14-16	+ 0.6	+ 2.6
Kitty Hawk Wind (60)	OCS-A 0508	59	1,000	>15 [c]	+ 0.4	+ 2.3
New England Wind[e y] (61)	OCS-A 0534	69	2,036 [b]	≥16	+ 0.3	+ 2.2
Beacon Wind (1 and 2) (62)	OCS-A 0520	79	1,230	8-20 [c]	+ 0.3 to + 0.6	+ 2.4 to + 2.8
SouthCoast Wind[f y] (63)	OCS-A 0521	88	804	12+ [c]	+ 0.2 to + 0.3	+ 2.2 to + 2.4

[a] Scores are shown as the suitability score difference described earlier.
[b] Outside the range of this analysis. We used the closest option.
[c] Not specified in project description but interpreted based on reported data.
[d] A second project (Ocean Wind 2: 1,148 MW) in the same lease area was also cancelled but had not submitted a COP yet (64).
[e] Formerly known as Vineyard Wind South. The project consists of two phases: Park City Wind (804 MW) and Commonwealth Wind (1,232 MW).
[f] Formerly known as Mayflower Wind.
[g] Site now falls in a De Facto Marine Protected Area. Provided score is the result before removing DMPAs from the analysis.
[x] Cancelled project in which development efforts by the project sponsors have ceased.
[y] Project withdrew from offtake agreement (PPA) and paid penalty to do so. There is no indication that the project will cease yet.

The result depicted in Figure 2 of the main text with the baseline facility—that near shore sites score lower under the developer paradigm than sites farther from shore—is also found here with various facilities. Low stakeholder scores are only found within 60km of the shore, while developer scores do not exhibit such a relationship. This behavior with stakeholder scores is unsurprising as, generally, proximate sites face more opposition from the public and other stakeholders (falling as much as 40% from near-shore to far-from shore sites) (65) and marine

biodiversity declines with depth (66)—both of which are metrics that inform the stakeholder weighting and scores.

Note 11. Detailed Methodology

We calculate suitability score with the following steps, with further detail given in Figure S9:

1. Analysis domain setup
2. Grid square metric calculations
3. Plant site metric calculations
4. Suitability score calculations

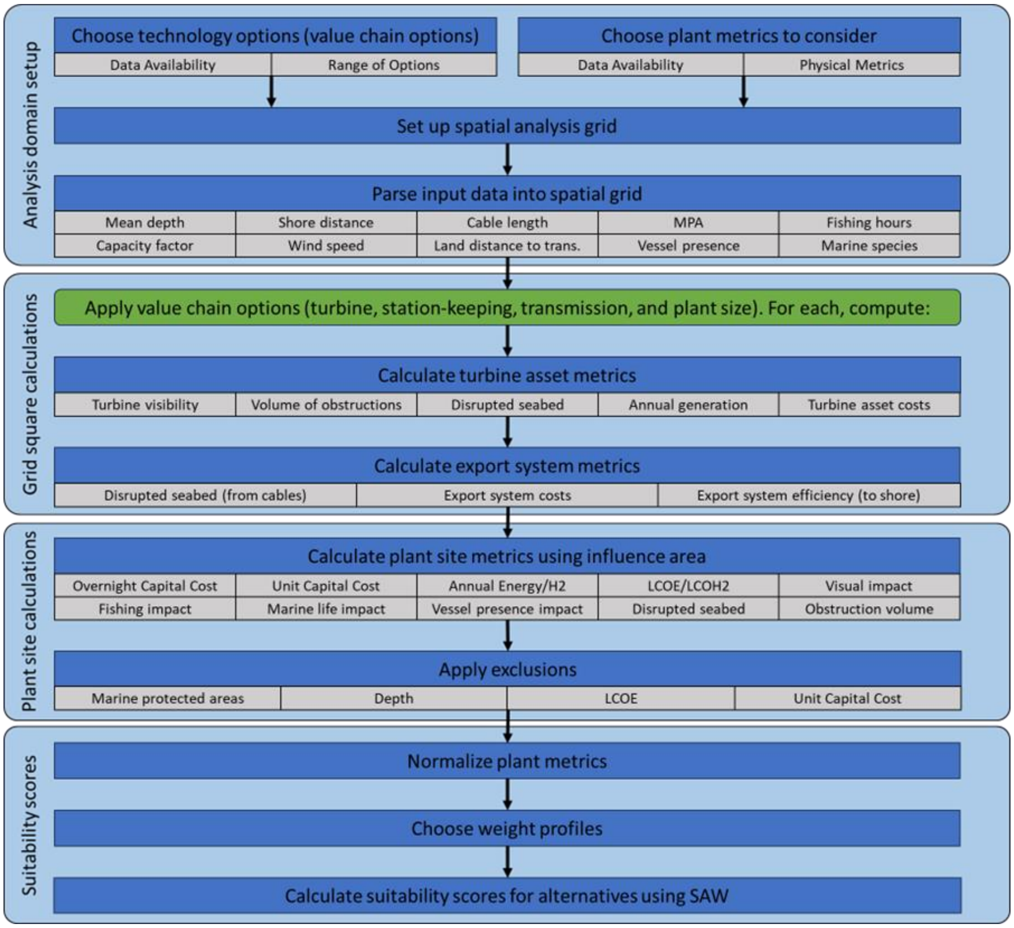

Figure S9. Suitability score methodology process.

Analysis domain setup. The technological domain describes the value chain options of turbine size, station-keeping type, plant size, and transmission method given in Table S14 below.

Table S14. Value chain options for the set of OSW plants considered in this paper.

Turbine size	Station-keeping method	Transmission method	Plant size
• 5 MW<br>• 8 MW<br>• 10 MW<br>• 15 MW	• 'Fixed': Fixed foundations using monopiles<br>• 'Moored': Floating semisubmersible foundations with mooring chains<br>• 'DP': Floating semisubmersible foundation with dynamic positioning (DP)	• 'HVC': High voltage export cable. Either alternating or direct current.<br>• 'PtH': Power-to-hydrogen (PtH) that is shipped to shore	• 200 MW<br>• 400 MW<br>• 600 MW<br>• 800 MW<br>• 1,000 MW<br>• 1,200 MW<br>• 1,400 MW
• 'No-Action': Alternative where no plant is built			

The metrics in Table S15 are collected and parsed into a value for each grid square; the source for each metric is provided in the table.

Table S15. Data for each grid square.

Parameter	Description	Units	Source
Mean depth	Mean water depth in the grid square	m	(67)
Distance to shoreline	Distance from centroid of grid square to the nearest shore	km	*
Length of submerged cables	Length of submarine cables within the grid square	m	(68)
Marine protected area	Whether the grid square overlaps with an established marine protected area (MPA)	[1,0]	(69)
Shipping fairways	Whether the grid square overlaps with an established shipping fairway.	[1,0]	(70–73)
De Facto marine protected area	Whether the grid square overlaps with a de facto marine protected area (DMPA) which is a place restricted by law for reasons other than conservation or natural resource management (includes natural and cultural heritage sites)	[1,0]	(74)
Fishing effort hours	Mean of maximum fishing vessel effort from 2012-2021	hrs	(75)
Capacity factor	Capacity factor for a turbine installed in the grid square	%	(76)
Mean wind speed	Mean wind speed in the grid square	m/s	(76)
Land distance to transmission system	Distance from landfall point to point of interconnection	km	(76)
Vessel traffic	Mean of the maximum number of transits from 2015-2021	vessels/ area	(77)
Marine life	Marine life species richness	species/ area	(78)

* Calculated using GIS software

The fishing effort value came from a finer grid (1.1 km x 1.1 km) than the one used here (75). We take the maximum value among the observations within each grid square as the value for the year. The same process was used for the number of vessel transits in each grid square in which the original dataset used a 100 m x 100 m grid (77).

Capacity factor, wind speed, and landfall distance to transmission were all calculated using a National Renewable Energy Laboratory (NREL) dataset (76) which was less granular than the grid spacing used (the spacing used was greater than 2 km x 2 km). The values of each metric in the nearest point in the NREL dataset to the centroid of each grid square was recorded as the value for each grid square.

The marine life value used data from the UNESCO Ocean Biodiversity Information System (78) and followed a data processing procedure by Provoost (79).

Grid square metric calculations. Further details on the calculation of the metrics listed in Table S16 are provided below.

Table S16. Additional grid square metrics computed for each combination of turbine size, station-keeping type, and transmission.

Metric	Units	Specific to Value Chain Option
Turbine visibility	[0,1]	
Obstructions	m^3/turbine	
Disrupted seabed	m^2/turbine or m^2/collector	Fixed, Moored, HVC
Turbine annual energy	MWh/turbine-year	
Turbine asset costs	MUSD/turbine	
Export system costs	MUSD/plant	
Export system efficiency	%	

Turbine visibility: The minimum object size that is discernable with normal visual acuity is one arc minute (80). Considering the tower width (which is the largest single body of the turbine), the distance at which the turbine tower is equal to one arc minute in an observer visual field (standing on the shore) is the sight distance used here. In other words, to be visible the tower diameter, D, must be greater than one arc minute of the circumference of a circle a distance r away:

$$D_{\text{tower}} \geq \frac{2\pi r}{360 \times 60}$$

For a given tower diameter, solving for r_{sight} gives the maximum distance at which a tower is discernible, this simplifies to,

$$r_{\text{sight}} \leq \frac{10{,}800 \times D_{\text{tower}}}{\pi}$$

Which becomes the necessary condition for which a single turbine is visible from shore. The results for the turbines in this study are reported in Table S17. If a grid square has a distance to shore less than r_{sight} for the chosen turbine, its visibility value is one, or else it is zero.

Table S17. Turbine sizes and sight distances.

Turbine Capacity (MW)	Turbine Tower Diameter, D_{tower} (m)	Computed Turbine Sight Distance, r_{sight} (km)
5	6 (10)	20.6
8	7.7 (11)	26.5
10	8.3 (12)	28.5
15	10 (13)	34.4

Obstructions: This metric is calculated as the volume of submerged substructure and submerged moorings. For monopiles, the volume of submerged substructure is estimated as the volume of a cylinder with a diameter equal to the base diameter of the turbine and a height equal to the mean water depth of the grid square. The volume of the submerged floating substructure is computed using the dimensions and specifications of the semisubmersible used (15). For moored floating turbines, the length of submerged mooring chains is calculated as (81):

$$L_{\text{s}} = h\sqrt{\left(2\frac{T_{\text{H}}}{\omega h} + 1\right)}$$

where h is water depth (m), T_{H} is the horizontal load (tons), and ω is the unit weight of the mooring chain (ton/m). We assume chains weigh between 0.1-0.6 ton/m (31) which have breaking loads greater than the design loads across all turbines. A submerged mooring chain is considered to have a swept volume of 1 m^3/m of submerged mooring chain (82).

Disrupted seabed: The disrupted seabed is computed for components that lie on the seafloor. These are: scour protection, mooring chains on the seafloor, and cables. For each fixed turbine, the area of scour protection needed considers a scour radius of 50 m (83). For a moored turbine, the area of disrupted seabed from mooring chains is computed following Qi Pan et al. (84) and considers three mooring chains. For the export cable, the minimum distance to shore from the collector is considered with a buffer on either side of 60 m (85). In practice, the length of the cable and the cable corridor path would be determined based on the location of the onshore substation, topology, and cable route. The latter can be a spatial multi-objective decision problem itself (86). Considering the minimum distance to shore, as we do here, gives a relative indication of the cable length, but should be seen as a lower-bound cable length for a plant.

Turbine annual energy: The annual output of a given turbine in a grid square is computed using the capacity factor and turbine size as,

$$\mathbb{E}_{\mathrm{turbine}}\left[\frac{\mathrm{MWh}}{\mathrm{year}}\right] = \mathrm{Cap\_factor} \times \mathrm{Turbine}[\mathrm{MW}] \times 8760\left[\frac{\mathrm{hrs}}{\mathrm{year}}\right]$$

when considering a DP turbine, the expected energy output is reduced by the thruster consumption percent (TCP, see SI Note 4). The energy output of a DP turbine is thus reduced by the TCP as,

$$\mathbb{E}_{\mathrm{DP\ turbine}}\left[\frac{\mathrm{MWh}}{\mathrm{year}}\right] = \mathbb{E}_{\mathrm{turbine}} \times \left(1 - \mathrm{TCP}(V_{\mathrm{wind}}, T)\right)$$

Where the TCP is a function of the mean wind speed, V_{wind}, and the turbine size, T.

Turbine asset costs: The sum of the total costs incurred by installing a turbine in a grid square is computed considering the cost of the following components:

- Turbine
- Monopile (fixed)
- Semisubmersible (moored, DP)
- Inter-array cable

Export system costs: The sum of the total costs incurred by installing the collector system in a grid square is computed considering the cost of the following components:

- Submarine export cable cost (HVC)
- Offshore substation costs (HVC)
- Electrolyzer cost (PtH)
- Hydrogen shipping vessel cost (PtH)
- Transformer cost

Export system efficiency: The efficiency of the export system is used to calculate the amount of energy delivered to shore annually. It is computed by considering the conversion efficiency of components at the collector site and the losses incurred in transit from the collector site to shore. For cable transmission, transformer efficiency, cable parameters, and functions to compute cable losses come from Xiang et al. (18). Resulting simplified equations for the efficiency of HVAC and HVDC transmission to shore are:

$$\eta_{\mathrm{AC,trans}} = 1 - \frac{S_{\mathrm{TT}} \times F \times \eta_{\mathrm{AC,offT}}}{V_{\mathrm{cn}}^2} \times \frac{r_{\mathrm{c}} \times l_{\mathrm{c}}}{\mathrm{nc}_{\mathrm{AC}}}$$

$$\eta_{\mathrm{DC,trans}} = 1 - \frac{S_{\mathrm{TT}} \times F \times \eta_{\mathrm{DC,offC}}}{V_{\mathrm{cn}}^2} \times \frac{r_{\mathrm{c}} \times l_{\mathrm{c}}}{2\mathrm{nc}_{\mathrm{DC}}}$$

where S_{TT} is the power rating of system (in megavolt-amps, MVA), F is the power factor for transmission (we assume this to be 1.0), $\eta_{\mathrm{AC,offT}}$ is the efficiency of the HVAC offshore transformer station (equal to 99.4%), $\eta_{\mathrm{DC,offC}}$ is the efficiency of the HVDC offshore converter station (equal to 98.28%), V_{cn} is the nominal voltage of the cable (kV), r_{c} is the cable resistance per kilometer (Ω/km), l_{c} is the length of the cable (km), nc is the number subsea cable circuits needed, computed as the active power transfer capability of one circuit over the needed active power transfer (size of the plant). This is computed using the functions in Xiang et al. (18) and considering several cable options provided in the SI Table S3-S4. For installations close to shore (< 80 km) the cable alternative considers an HVAC transmission system, while for far-shore installations (80 km and beyond) HVDC is the cable technology used (87). The resulting efficiency, cost, and cable space metrics are computed considering this cable transmission option.

For PtH transmission, electrolyzer efficiency, liquefication efficiency, and losses from burn-off (storage) in transit come from various sources (see SI Table S8). The resulting export efficiency is expressed as a percent for each plant alternative which can then be used to calculate the amount of energy delivered to shore annually—the annual energy delivered to shore is the product of the energy produced by the OSW plant and the export system efficiency. Both export systems incur greater losses with greater distance from shore.

Plant site metric calculation. Following the procedure outlined in the main text, the plant metrics described in Table S18 below are calculated.

Table S18. Attributes employed in the spatial multi-criteria analysis**.**

Plant metric	Main Interested Party	Type	Description	Unit
Overnight Capital Cost	Developer	Cost	Estimated Capital Cost of fabrication and acquisition of the plant	MUSD
Unit Overnight Capital Cost	Developer	Cost		USD/MW
Annual Energy/H_2 Output	Developer, Public	Benefit	Expected annual energy output (electricity or hydrogen)	MWh/year or tH_2/year
LCOE/LCOH	Developer	Cost	Levelized cost of energy or levelized cost of hydrogen	USD/MWh or USD/kgH_2
Visual Impact	Coastal Residents	Cost	Expected number of visible turbines from shore	Turbines/plant
Impact on Fishing	Fisheries	Cost	Expected hours of fishing effort in the installed plant area (pre-plant existence)	Hours/year
Impact on Marine Life	Environmentalists, Conservationists	Cost	Expected number of observed species in the installed plant area (pre-plant existence)	Species/plant
Impact on Vessel Traffic	Vessel Operators	Cost	Expected number of vessel transits in the installed plant area (pre-plant existence)	Vessel transits/plant
Disrupted Seabed	Environmentalists, Conservationists	Cost	Expected area of seabed disrupted or unusable in the installed plant area	km^2 of seabed/plant
Obstructions	Environmentalists, Conservationists, Fisheries	Cost	Expected volume of submerged obstructions in the installed plant area	m^3 of obstructions/ plant

To make these calculations, an Influence Area (IA) is considered. Figure S10 below illustrates an example influence area for a plant alternative with 10 turbines that shows how several plausible

orientations may fit within the IA. The metrics in Table S19 are computed for each plant alternative considering the IA, described in detail below.

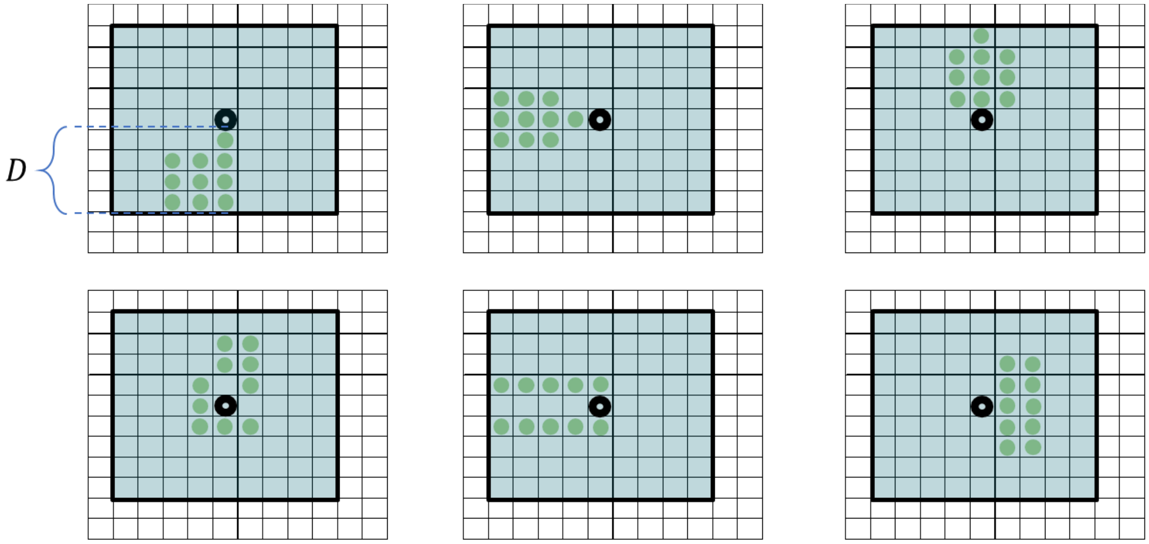

Figure S10. Illustration of the influence area given a collector site (black circle) for a 10-turbine plant. The influence area is the shaded area, and possible turbine locations are shown as green dots.

Table S19. No-Action decision suitability scores under different weighting paradigms. The maximum possible score under each set of weights is equal to 10. Symbolic representations of the variables are provided.

Metric	Cost or Benefit	No-Action Metric Value	Normalized No-Action Metric [a]	Uniform Weight	Developer Weight	Stakeholder Weight	High Concern	Low Concern
j	–	$x_{\mathrm{NA,j}}$	$Z_{\mathrm{NA,j}}$	w_{j}				
Overnight Capital Cost	Cost	0	1	1	2.5	0	0	0
Unit Overnight Capital Cost	Cost	0	1	1	2.5	0	0	0
Annual Energy/H_2 Output	Benefit	0	0	1	2.5	10/7	4	0.5
LCOE/LCOH	Cost	— [b]	0	1	2.5	0	0	0
Visual Impact	Cost	0	1	1	0	10/7	1	9.5/6
Impact on Fishing	Cost	0	1	1	0	10/7	1	9.5/6
Impact on Marine Life	Cost	0	1	1	0	10/7	1	9.5/6
Impact on Vessel Traffic	Cost	0	1	1	0	10/7	1	9.5/6
Disrupted Seabed	Cost	0	1	1	0	10/7	1	9.5/6
Obstructions	Cost	0	1	1	0	10/7	1	9.5/6
**No-Action Suitability Score (out of 10 possible): $\sum w_{\mathrm{j}} Z_{\mathrm{NA,j}}$**				**8.0**	**5.0**	**8.6**	**6.0**	**9.5**

[a] The normalized metrics are computed as defined in equation (2) of the main text considering if the metric is a cost or benefit.
[b] Undefined

Overnight Capital Cost and Unit Overnight Capital Cost: The capital costs of the components are summed to get a plant overnight capital cost, reported as the overall cost and cost per installed MW of plant capacity. This cost does not include certain project execution costs and assumes that the plant is built 'overnight', focusing on fabrication and acquisition costs. As shown in the SI Table S7, this method captures the relative differences between plant proposals when compared to NREL model results.

Annual Energy/H_2 Output: The energy output to shore is estimated considering the losses in transmission to shore and conversion (in the case of hydrogen production) as described earlier.

$$\mathbb{E}[\text{Annual Energy}] = \frac{\sum_{\text{IA}} \text{Annual turbine generation}}{\text{effective plants}} \times \text{transmission efficiency}$$

$$\mathbb{E}[\text{Annual H2}] = \frac{\sum_{\text{IA}} \text{Annual turbine generation}}{\text{effective plants}} \times \text{conversion and shipping efficiency}$$

LCOE/LCOH: The levelized cost of energy (LCOE) and levelized cost of hydrogen (LCOH) are computed as the net present value (NPV) of costs divided by the NPV of energy flows using the following equations.

$$LCOE\left[\frac{USD}{MWh}\right] = \frac{\text{NPV of costs}}{\text{NPV of energy flows}} = \frac{\sum_t \left(\frac{CAPEX_t + OPEX_t}{(1+r)^t}\right)}{\sum_t \left(\frac{\mathbb{E}[\text{Annual Energy}]}{(1+r)^t}\right)}$$

$$LCOH\left[\frac{USD}{MWh}\right] = \frac{\text{NPV of costs}}{\text{NPV of H2 flows}} = \frac{\sum_t \left(\frac{CAPEX_t + OPEX_t}{(1+r)^t}\right)}{\sum_t \left(\frac{\mathbb{E}[\text{Annual H2}]}{(1+r)^t}\right)}$$

These calculations consider overnight capital expenditure (CAPEX) and annual operational expenditures (OPEX) which are estimated as a percentage of CAPEX incurred per year, (see SI Table S7). A discount rate, r, equal to 5.2% is used (24).

Visual Impact: The expected number of visible turbines from shore is divided by the number of effective plants.

$$\text{Visual Impact} = \frac{\text{Visible turbines in influence area}}{\text{effective plants}}$$

Impact on Vessel Traffic: The expected vessel operations impact is estimated using the number of vessel transits in the IA before the plant is built divided by the number of effective plants. The measure used here is vessel transits in each grid square. Therefore, the same vessel transiting adjacent grid squares in the same IA is counted more than once. While this may seem like multiple counting of a single ship, in fact, it captures the intensity of the impact on traffic due to the development in the IA. For example, a vessel that transits only one grid square on the edge of the IA would be less impacted than a ship that transits through the middle of the IA, crossing many squares.

$$\text{Impact on Vessel Traffic} = \frac{\sum_{IA} \text{Vessel transits}}{\text{effective plants}}$$

Impact on Fishing: The expected hours of fishing effort per year that took place prior to building the plant in the IA are considered as the impact on fishing efforts. This is estimated using the hours of fishing effort in the IA divided by the number of effective plants. The same rationale to address potential ‘multiple counting’ for the impact on vessel traffic applies here.

$$\text{Impact on Fishing} = \frac{\sum_{IA} \text{Hours of fishing}}{\text{effective plants}}$$

Impact on Marine Life: The expected impact on marine life is quantified as the number of species observed in the IA divided by the number of effective plants. The same rationale to address potential ‘multiple counting’ for the impact on vessel traffic applies here.

$$\text{Impact on Marine Life} = \frac{\sum_{IA} \text{Marine species richness}}{\text{effective plants}}$$

Disrupted Seabed: The total area of disrupted seabed expected is estimated as the disrupted seabed caused by the turbines and cabled transmission systems.

$$\text{Disrupted seabed} = \frac{\sum_{IA} \text{Disrupted seabed from turbines}}{\text{effective plants}} + \text{Disrupted seabed from export}$$

Obstructions: The sum of the obstructions from the turbine installations is considered as the overall plant obstructions.

$$\text{Obstructions} = \frac{\sum_{\text{IA}} \text{Obstructions}}{\text{effective plants}}$$

SI References

1. Navigant Consulting Inc., “Offshore Wind Market and Economic Analysis 2014 Annual Market Assessment” (2014).
2. Navigant Consulting Inc., “Offshore Wind Market and Economic Analysis Annual Market Assessment” (2013).
3. Navigant Consulting Inc., “Offshore Wind Market and Economic Analysis Annual Market Assessment” (2013).
4. W. Musial, *et al.*, “2016 Offshore Wind Technologies Market Report” (2016).
5. P. Beiter, *et al.*, “2017 Offshore Wind Technologies Market Update” (2018).
6. U.S. DOE, “2018 Offshore Wind Technologies Market Report - Executive Summary” (2018).
7. W. Musial, *et al.*, “Offshore Wind Market Report: 2021 Edition” (2021).
8. W. Musial, *et al.*, “Offshore Wind Market Report: 2022 Edition” (2022).
9. 4COffshore, Global Offshore Wind Farms Database | 4C Offshore. (2023). Available at: https://www.4coffshore.com/windfarms/ [Accessed 12 May 2023].
10. J. Jonkman, S. Butterfield, W. Musial, G. Scott, “Definition of a 5-MW reference wind turbine for offshore system development” (2009).
11. C. Desmond, J. Murphy, L. Blonk, W. Haans, Description of an 8 MW reference wind turbine. *J Phys Conf Ser* **753** (2016).
12. P. Bortolotti, *et al.*, “IEA Wind Task 37 on Systems Engineering in Wind Energy - WP2.1 Reference Wind Turbines: Technical Report” (2019).
13. E. Gaertner, *et al.*, “Definition of the IEA Wind 15-Megawatt Offshore Reference Wind Turbine Technical Report” (2020).
14. IEA, “Offshore Wind Outlook 2019” (2019).
15. C. Allen, *et al.*, “Definition of the UMaine Volturn US-S Reference Platform Developed for the IEA Wind 15-Megawatt Offshore Reference Wind Turbine” (2020).
16. S. Xu, M. Murai, X. Wang, K. Takahashi, A novel conceptual design of a dynamically positioned floating wind turbine. *Ocean Engineering* **221**, 108528 (2021).
17. P. Connolly, C. Crawford, Analytical modelling of power production from Un-moored Floating Offshore Wind Turbines. *Ocean Engineering* **259**, 111794 (2022).
18. X. Xiang, *et al.*, Comparison of cost-effective distances for LFAC with HVAC and HVDC in their connections for offshore and remote onshore wind energy. *CSEE Journal of Power and Energy Systems* **7**, 954–975 (2021).
19. A. N. Alkhaledi, S. Sampath, P. Pilidis, Propulsion of a hydrogen-fuelled LH2 tanker ship. *Int J Hydrogen Energy* **47**, 17407–17422 (2022).
20. A. N. Alkhaledi, S. Sampath, P. Pilidis, Economic analysis of a zero-carbon liquefied hydrogen tanker ship. *Int J Hydrogen Energy* (2022). https://doi.org/10.1016/J.IJHYDENE.2022.06.168.
21. R. Santarromana, *et al.*, Assessing the costs and benefits of dynamically positioned floating wind turbines to enable expanded deployment. *Energy Convers Manag* **306**, 118301 (2024).
22. ABS, “Guide for Building and Classifying Floating Offshore Wind Turbine Installations” (2013).
23. ABS, “Guide for Dynamic Positioning Systems” (2020).
24. NREL, Offshore Wind Annual Technology Baseline. *Annual Technology Baseline: Offshore Wind* (2021). Available at: https://atb.nrel.gov/electricity/2021/offshore_wind [Accessed 5 November 2021].
25. P. Beiter, *et al.*, “The Cost of Floating Offshore Wind Energy in California Between 2019 and 2032” (2020).
26. A. Myhr, C. Bjerkseter, A. Ågotnes, T. A. Nygaard, Levelised cost of energy for offshore floating wind turbines in a lifecycle perspective. *Renew Energy* **66**, 714–728 (2014).
27. P. Beiter, *et al.*, “A Spatial-Economic Cost-Reduction Pathway Analysis for U.S. Offshore Wind Energy Development from 2015–2030” (2016).

28. G. E. Jung, M. C. Dinh, H. J. Sung, M. Park, I. K. Yu, Economic analysis of a 22.9 kv hts power cable and conventional ac power cable for an offshore wind farm connections. *Progress in Superconductivity and Cryogenics (PSAC)* **20**, 60–64 (2018).
29. F. H. Saadi, N. S. Lewis, E. W. McFarland, Relative costs of transporting electrical and chemical energy. *Energy Environ Sci* **11**, 469–475 (2018).
30. T. Van Wingerden, D. Geerdink, C. Taylor, C. F. Hülsen, "Specification of a European Offshore Hydrogen Backbone" (2023).
31. Daihan Anchor Chain Mfg. Co. Ltd., Proof & Breaking Loads for Studless Link Mooring Chain (I). (2020). Available at: http://www.dhac.co.kr/m32.php?pn=3&sn=2&sn2=1 [Accessed 11 November 2020].
32. N. Avanessova, A. Gray, I. Lazakis, R. C. Thomson, G. Rinaldi, Analysing the effectiveness of different offshore maintenance base options for floating wind farms. *Wind Energy Science* **7**, 887–901 (2022).
33. Vice President (Personal Communication), Kongsberg Thruster Specifications. [Preprint] (2021).
34. Director (Personal Communication), Kongsberg O&M Estimates. [Preprint] (2022).
35. Unites States Coast Guard, "The Areas Offshore of Massachusetts and Rhode Island Port Access Route Study" (2020).
36. X. Xiang, M. M. C. Merlin, T. C. Green, Cost analysis and comparison of HVAC, LFAC and HVDC for offshore wind power connection. *IET Conference Publications* **2016** (2016).
37. S. M. Saba, M. Müller, M. Robinius, D. Stolten, The investment costs of electrolysis – A comparison of cost studies from the past 30 years. *Int J Hydrogen Energy* **43**, 1209–1223 (2018).
38. IRENA, "Green Hydrogen Cost Reduction: Scaling Up Electrolysers to meet the 1.5C Climate Goal" (2020).
39. S. P. Popov, O. A. Baldynov, Evaluation of energy efficiency of the long distance energy transport systems for renewable energy. *Energy Systems Research* (2019). https://doi.org/10.1051/e3sconf/20191140.
40. R. M. Dawes, B. Corrigan, "Linear Models in Decision Making" (1974).
41. R. M. Dawes, The robust beauty of improper linear models in decision making. *American Psychologist* **34**, 571–582 (1979).
42. C. Wilson, *et al.*, Granular technologies to accelerate decarbonization. *Science (1979)* **368**, 36–39 (2020).
43. A. Malhotra, T. S. Schmidt, Accelerating Low-Carbon Innovation. *Joule* **4**, 2259–2267 (2020).
44. B. Sweerts, R. J. Detz, B. van der Zwaan, Evaluating the Role of Unit Size in Learning-by-Doing of Energy Technologies. *Joule* **4**, 967–970 (2020).
45. D. Caporale, C. De Lucia, Social acceptance of on-shore wind energy in Apulia Region (Southern Italy). *Renewable and Sustainable Energy Reviews* **52**, 1378–1390 (2015).
46. J. Meyerhoff, Do turbines in the vicinity of respondents' residences influence choices among programmes for future wind power generation? *Journal of Choice Modelling* **7**, 58–71 (2013).
47. A. Gumber, R. Zana, B. Steffen, A global analysis of renewable energy project commissioning timelines. *Appl Energy* **358**, 122563 (2024).
48. BOEM, Cape Wind. (2017). Available at: https://www.boem.gov/renewable-energy/studies/cape-wind [Accessed 15 February 2023].
49. M. Serreze, Commercial fisheries sue Biden administration over Vineyard Wind I. *Providence Business First* (2022). Available at: https://www.bizjournals.com/rhodeisland/news/2022/02/01/roda-sues-feds-over-vineyard-wind.html [Accessed 10 July 2022].
50. US Wind, "Construction and Operations Plan: Maryland Offshore Wind Project" (2023).
51. Atlantic Shores Offshore Wind, "Atlantic Shores [North] Offshore Wind - Construction and operations Plan" (2023).
52. Ocean Wind LLC, "Construction & Operations Plan Ocean Wind Offshore Wind Farm" (2023).

53. Atlantic Shores Offshore Wind, “Atlantic Shores [South] Offshore Wind - Construction and Operations Plan” (2023).
54. Revolution Wind, “Construction & Operations Plan Revolution Wind Farm Volume I” (2023).
55. Equinor, “Empire Wind Project (EW 1 and EW 2) Construction and Operations Plan Volume 1: Project Information” (2022).
56. South Fork Wind, “South Fork Construction and Operations Plan” (2021).
57. Vineyard Wind, “Draft Construction and Operations Plan Volume I” (2020).
58. Sunrise Wind, “Sunrise Wind Farm Project Construction and Operations Plan” (2022).
59. Dominion Energy, “Construction and Operations Plan: Coastal Virginia Offshore Wind Commercial Project” (2023).
60. Kitty Hawk Wind LLC, “Construction and Operations Plan - Executive Summary” (2022).
61. Park City Wind LLC, “New England Wind Construction and Operations Plan for Lease Area OCS-A 0534 Volume I Text” (2022).
62. Beacon Wind LLC, “Beacon Wind Project: Beacon Wind 1 and Beacon Wind 2 Construction and Operations Plan” (2023).
63. Mayflower Wind, “Construction and Operations Plan Volume 1” (2022).
64. Orsted, Orsted Ceases Development of Ocean Wind 1 and Ocean Wind 2. (2023). Available at: https://oceanwindone.com/news-archive/2023/11/orsted [Accessed 16 January 2024].
65. W. Musial, D. Heimiller, P. Beiter, G. Scott, C. Draxl, “2016 Offshore Wind Energy Resource Assessment for the United States” (2016).
66. M. J. Costello, C. Chaudhary, Marine Biodiversity, Biogeography, Deep-Sea Gradients, and Conservation. *Current Biology* **27**, R511–R527 (2017).
67. GEBCO Bathymetric Compilation Group 2020, The GEBCO_2020 Grid - a continuous terrain model of the global oceans and land. (2020).
68. Data.gov, Submarine Cable Lines (USACE IENC) - Catalog. (2022). Available at: https://catalog.data.gov/dataset/submarine-cable-lines-usace-ienc [Accessed 22 May 2023].
69. NOAA National Marine Protected Areas Center, The MPA Inventory. (2020). Available at: https://marineprotectedareas.noaa.gov/dataanalysis/mpainventory/ [Accessed 30 September 2022].
70. Shipping safety fairways and anchorage areas, Gulf of Mexico.
71. Areas along the coast of California.
72. Areas along the coast of Alaska.
73. Areas along the Atlantic Coast.
74. N. O. and A. A. US Department of Commerce, De Facto Marine Protected Areas. *National Marine Protected Areas Center* (2023). Available at: https://marineprotectedareas.noaa.gov/dataanalysis/defacto/ [Accessed 24 January 2024].
75. Global Fishing Watch, Data download portal. (2022). Available at: https://globalfishingwatch.org/data-download/ [Accessed 13 June 2022].
76. NREL, The Wind Prospector. (2020). Available at: https://maps.nrel.gov/?da=wind-prospector [Accessed 11 December 2020].
77. MarineCadastre.gov, Data Registry. (2023). Available at: https://marinecadastre.gov/data/ [Accessed 22 May 2023].
78. OBIS, Ocean Biodiversity Information System - Data Access. (2022). Available at: https://obis.org/data/access/# [Accessed 10 January 2023].
79. P. Provoost, Diversity indicators using OBIS data. (2021). Available at: https://iobis.github.io/notebook-diversity-indicators/ [Accessed 22 December 2022].
80. N. Maslov, C. Claramunt, T. Wang, T. Tang, Method to estimate the visual impact of an offshore wind farm. *Appl Energy* **204**, 1422–1430 (2017).
81. W. H. Huang, R. Y. Yang, Water depth variation influence on the mooring line design for fowt within shallow water region. *J Mar Sci Eng* **9** (2021).
82. V. Harnois, H. C. M. Smith, S. Benjamins, L. Johanning, Assessment of entanglement risk to marine megafauna due to offshore renewable energy mooring systems. *International Journal of Marine Energy* **11**, 27–49 (2015).

83. ICF, “Comparison of Environmental Effects from Different Offshore Wind Turbine Foundations” (2020).
84. Q. Pan, M. Y. Mahfouz, F. Lemmer, Assessment of mooring configurations for the IEA 15MW floating offshore wind turbine. *J Phys Conf Ser* 12030 (2018). https://doi.org/10.1088/1742-6596/2018/1/012030.
85. US DOI - Bureau of Safety and Environmental Enforcement, “Offshore Wind Submarine Cable Spacing Guidance” (2014).
86. D. Engberg, J. Cohon, C. ReVelle, Multiobjective modeling for OCS Pipeline Systems in *The Second Symposium on Management, Conservation and Utilization of the Coastal Zone*, (1980).
87. K. Samoteskul, J. Firestone, J. Corbett, J. Callahan, Changing vessel routes could significantly reduce the cost of future offshore wind projects. *J Environ Manage* **141**, 146–154 (2014).
